# A LAMP–CRISPR/Cas12a Assay for Specific Detection of *Escherichia coli* in Whole Blood: Analytical Performance and Clinical Evaluation in Suspected Bloodstream Infection

**DOI:** 10.3390/diagnostics16213692

**Published:** 2026-11-09

**Authors:** Dao Thanh Quyen, Nguyen Sy Thau, Pham Quang Trung, Nguyen Quang Huy, Dao Thi Huyen, Phan Quoc Hoan, Do Thu Trang, Le Mai Parkes, Le Huu Song, Ngo Tat Trung

**Affiliations:** 1 Vietnamese-German Center for Medical Research, 108 Military Central Hospital, Tran Hung Dao Street N1, Hai Ba Trung, Hanoi 100000, Vietnam; quyendao108@gmail.com (D.T.Q.); daohuyen.med@gmail.com (D.T.H.); songlh@benhvien108.vn (L.H.S.); 2 University of Science and Technology of Hanoi (USTH), Vietnam Academy of Science and Technology (VAST), 18 Hoang Quoc Viet, Hanoi 100000, Vietnam; nguyen-quang.huy@usth.edu.vn; 3 Institute of Clinical Infectious Diseases, 108 Military Central Hospital, Tran Hung Dao Street N1, Hai Ba Trung, Hanoi 100000, Vietnam; nguyensythau0211@gmail.com; 4 Department of Molecular Biology, 108 Military Central Hospital, Tran Hung Dao Street N1, Hai Ba Trung, Hanoi 100000, Vietnam; trungmeoww@gmail.com (P.Q.T.); phan_hoan2002@yahoo.com (P.Q.H.); 5 Department of Microbiology, 108 Military Central Hospital, Tran Hung Dao Street N1, Hai Ba Trung, Hanoi 100000, Vietnam; dr.thutrang1709@gmail.com; 6 Division of Informatics, Imaging and Data Sciences, The University of Manchester, Manchester M13 9PL, UK; maisu10193@gmail.com

**Keywords:** bloodstream infection, sepsis, *Escherichia coli*, loop-mediated isothermal amplification, CRISPR/Cas12a, limit of detection, probit regression, point-of-care testing

## Abstract

**Background/Objectives:** Blood culture is slow and real-time PCR (qPCR) needs a thermal cycler, while loop-mediated isothermal amplification (LAMP) is rapid but prone to non-specific amplification. We developed a LAMP assay with CRISPR/Cas12a read-out targeting the *Escherichia coli dxs* gene and compared it with qPCR and blood culture. **Methods:** Reaction conditions were optimised and specificity tested on 15 *E. coli* and 40 non-target isolates. The limit of detection (LOD) was determined in spiked whole blood (20 replicates per level, 1–10 CFU/mL) by hit rate and probit regression, with conversion to *dxs* copies by growth phase. Fifty-two patients with suspected bloodstream infection were tested by all three methods. **Results:** The assay detected all 15 *Escherichia coli* isolates tested (inclusivity 15/15; 95% CI 79.6–100) and none of 40 non-target isolates (exclusivity 40/40; 95% CI 91.2–100); the exclusivity panel did not include *Shigella* spp. or *Escherichia fergusonii*, which the in silico analysis predicts the assay would detect. The probit LOD_95_ was 14.2 CFU/mL (95% fiducial limits 6.7–156; ≈43 *dxs* copies/mL for the mid-exponential spike culture used here) for LAMP-CRISPR/Cas12a and 5.8 CFU/mL (2.6–958) for qPCR; a positive was read within 5 min of starting the Cas12a detection step. LAMP-CRISPR/Cas12a was positive in 19/33 qPCR-positive and 0/19 qPCR-negative patients (positive and negative percent agreement 57.6% and 100%; *κ* = 0.50); all 13 samples at or above the qPCR LOD_95_ were detected. **Conclusions:** LAMP-CRISPR/Cas12a detects *E. coli* in whole blood specifically and rapidly, but its clinical sensitivity is limited by the very low bacterial loads of bacteraemia, so that it is suited to use as a rapid confirmatory test alongside blood culture rather than as a rule-out test.

## 1. Introduction

Sepsis is life-threatening organ dysfunction caused by a dysregulated host response to infection [1]. It affects about 49 million people and causes 11 million deaths each year, roughly one in five deaths worldwide [2,3]. In septic shock, each hour of delay in effective antimicrobial therapy increases mortality [4], so rapid identification of the causative organism matters.

Blood culture remains the reference method for bloodstream infection (BSI), and culture-based recovery followed by biochemical or mass-spectrometric identification is still the basis of microbiological diagnosis [5], but it has well-known limitations. It needs 24–72 h of growth [6], loses sensitivity after antibiotic exposure and for fastidious organisms [7,8], requires 20–30 mL of blood per set [8,9], and is positive in only 10–20% of sets [10]. Part of the problem is biological: most adults with bacteraemia carry fewer than 10 CFU/mL of blood, and many fewer than 1 CFU/mL [11], but they are affected by inhibitors and the excess of human DNA in blood [12,13] and require thermal cyclers and trained staff, which limits their use outside central laboratories.

*Escherichia coli* is the leading Gram-negative cause of BSI [14]. It was the most frequent cause of early-onset neonatal sepsis in a cohort of 217,480 newborns [15], it predominates among Gram-negative isolates from septic patients in Vietnam [16], and it is the main pathogen in urosepsis, where in-hospital mortality still reaches 11% [17]. A rapid, specific *E. coli* test that works directly on blood would therefore be clinically useful.

Loop-mediated isothermal amplification (LAMP) amplifies DNA at a single temperature within 30–60 min, but its four to six primers readily form non-specific products that dye- or turbidity-based read-outs score as positive. Coupling LAMP to the *trans*-cleavage activity of Cas12a adds a sequence-specific recognition step: only amplicons that match the crRNA activate Cas12a and cleave a fluorescent reporter [18]. Reaction additives such as betaine, polyethylene glycol, trehalose and bovine serum albumin (BSA) can further improve LAMP efficiency in complex matrices [19,20,21], and Cas12a activity depends on metal ions, pH and stabilisers, and guide engineering can enhance activation [22,23,24,25].

Analytical sensitivity of blood assays is usually expressed in CFU/mL of a spiked culture. Because a growing *E. coli* cell carries several partially replicated chromosomes, the number of target copies per CFU depends on the growth phase of the culture, which complicates comparison between studies. Here we describe a LAMP-CRISPR/Cas12a assay targeting the *E. coli dxs* gene, determine its specificity and LOD in whole blood by hit rate and probit regression, express the LOD in *dxs* copies according to growth phase, and compare it with qPCR and blood culture in 52 patients with suspected BSI.

The assay is intended as a rapid, highly specific confirmatory test for *E. coli* in patients with suspected bloodstream infection, to be used alongside blood culture rather than as a test that can exclude infection.

## 2. Materials and Methods

### 2.1. Primer and crRNA Design

LAMP primers and a crRNA were designed using a conserved region of the *dxs* gene, encoding 1-deoxy-D-xylulose-5-phosphate synthase, in *E. coli* K-12 MG1655 (GenBank accession NC_000913.3; complement 438,315–440,177). The selected design region spanned positions 438,545–439,194, yielding a 243 bp F3–B3 amplicon (positions 438,921–439,163). For compatibility with wild-type LbCas12a, the crRNA target was selected to satisfy the canonical 5′-TTTV-3′ PAM requirement (V = A, C, or G) [18,26,27] and to lie between the F1 and B1 sites within the stem of the LAMP dumbbell structure. Two candidate protospacers met these criteria, and one was selected for the final assay. As the *dxs* region is conserved in both *E. coli* and *Shigella* spp., the assay targets the *E. coli/Shigella* clade rather than *E. coli* sensu stricto. Details of the sequence design and in silico validation are provided in Supplementary Methods S1 [18,28,29]. The qPCR primers and probe targeting *dxs* have been described previously [30,31]. All oligonucleotides are listed in Table 1.

### 2.2. Bacterial Strains, Spiked Whole Blood and DNA Extraction

*E. coli* K-12 MG1655 was grown in LB broth to OD_600_ 0.400, corresponding to 2.8 × 10^8^ CFU/mL by plate count, diluted 1:280 to 10^6^ CFU/mL and then ten-fold to 10^2^ CFU/mL in LB. Ten microlitres of each dilution were spiked into 990 µL of whole blood to give 10^4^, 10^3^, 10^2^, 10^1^ and 10^0^ CFU/mL; 10 µL of LB in blood served as the human DNA control, an *E. coli*-free whole-blood sample carried through the identical workflow (Figure S5). For spiked and clinical samples, 1 mL of whole blood underwent host-leukocyte depletion using MCLB-1 solution according to the published procedure [13], with the sole modification that the pellet was resuspended in buffer BL2 from the SaMag Bacterial DNA Extraction Kit (CE-IVD; Cat. No. SM006; Sacace Biotechnologies Srl, Como, Italy) and extracted using the kit according to the manufacturer’s instructions, rather than resuspended in 1× PBS and extracted manually with NaOH/SDS. DNA was eluted in 100 µL, of which 5 µL was used per reaction; assuming complete recovery, this corresponds to 0.05 CFU per reaction for a blood concentration of 1 CFU/mL. These volumes were kept constant across all replicates to ensure consistency in hit-rate and probit analyses. The mean DNA concentration was 32.8 ± 1.47 ng/µL, with an A260/280 ratio of 2.08 ± 0.04.

### 2.3. Real-Time PCR

qPCR targeting *dxs* was performed in 20 µL containing 1× QuantiTect Probe PCR Master Mix (Qiagen, Hilden, Germany), 0.25 µM of each primer, 0.25 µM probe and 5 µL template on an AriaMx (Agilent Technologies, Santa Clara, CA, USA) or CFX96 (Bio-Rad, Hercules, CA, USA) real-time PCR instrument: 95 °C for 15 min, then 45 cycles of 95 °C for 15 s and 60 °C for 60 s [13] with 95% confidence intervals (CI) from the slope t-interval, and 90–110% was taken as the acceptable range [33].

### 2.4. LAMP–CRISPR/Cas12a Reaction

**Figure 1:**
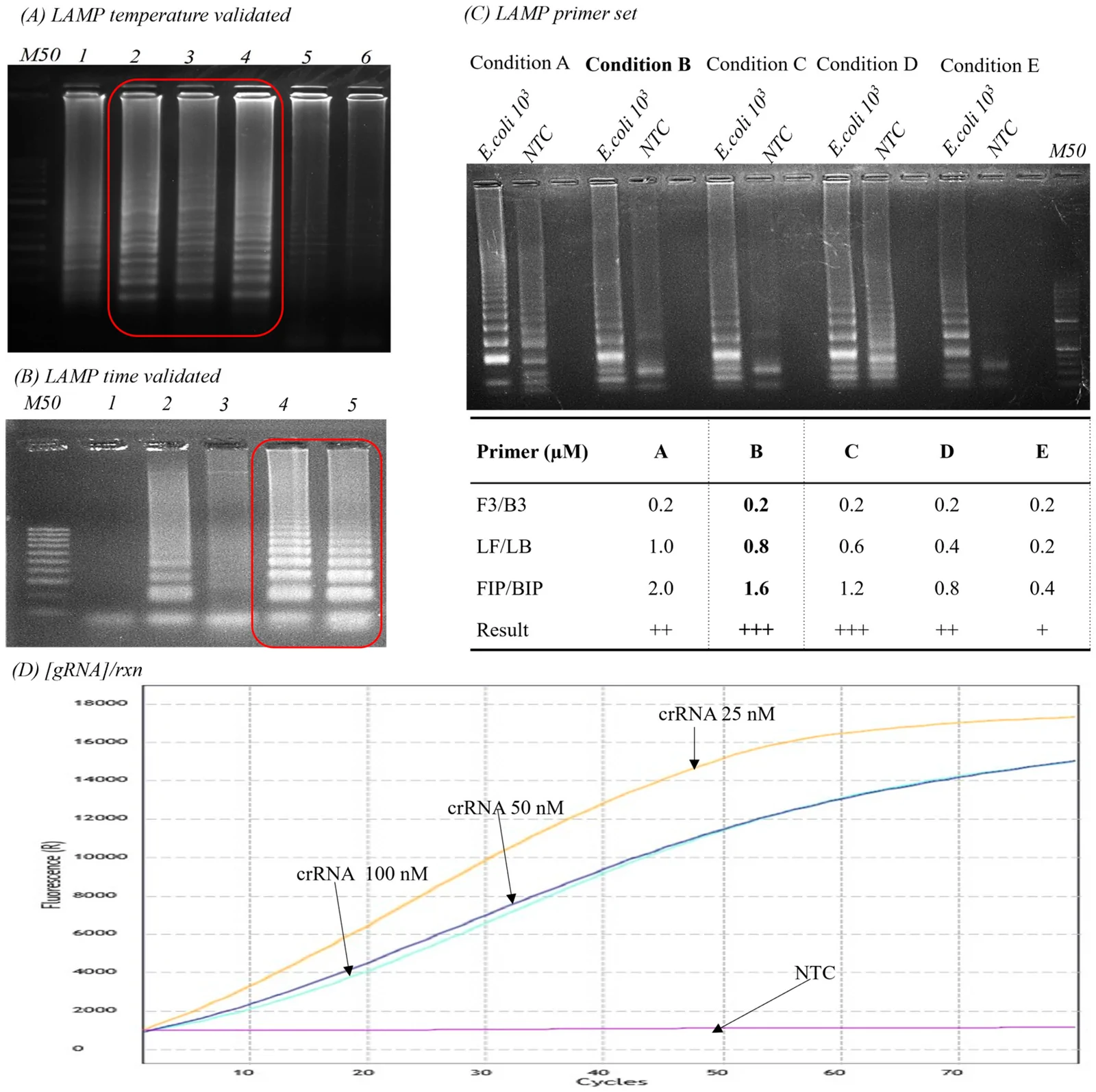
Optimisation of LAMP and CRISPR/Cas12a reaction conditions. (**A**) LAMP with *E. coli* template (10^3^ CFU/mL) at 59, 61, 63, 65 and 67 °C (lanes 1–5); lane 6, NTC. (**B**) LAMP reaction time: lanes 2–5, 30, 20, 40 and 60 min (not in ascending lane order); lane 1, NTC. (**C**) LAMP primer concentrations (conditions A–E), each with *E. coli* template and an NTC in adjacent lanes and separated by unloaded lanes; F3/B3 was 0.2 µM throughout. Products were ranked on a three-level scale: +, partial ladder or smear (three or fewer discrete bands, or continuous fluorescence without discrete bands); ++, ladder (more than three discrete bands with high-molecular-weight material at or near the well) accompanied by smear, or with a product also present in the no-template control; +++, ladder with no smear and no product in the no-template control. Condition B, 0.2 µM F3/B3, 1.6 µM FIP/BIP and 0.8 µM LF/LB, was selected and run at 65 °C for 40 min. (**D**) Effect of crRNA concentration (25, 50 and 100 nM per reaction) on Cas12a *trans*-cleavage fluorescence, with an NTC. Products were analysed on 2% agarose gels; M50, 50 bp ladder.

LAMP was performed with Bst 3.0 DNA polymerase (New England Biolabs, Ipswich, MA, USA) in 25 µL of 1× reaction buffer (20 mM Tris–HCl, 10 mM (NH_4_)_2_SO_4_, 150 mM KCl, 2 mM MgSO_4_, 0.1% Tween 20, pH 8.8) containing 200 U/mL Bst 3.0, 400 µM dNTPs (Thermo Scientific, Thermo Fisher Scientific, Vilnius, Lithuania), 100 µg/mL BSA per reaction (Molecular Biology Grade; New England Biolabs, Ipswich, MA, USA), with no additional MgSO_4_, and the primer set (Integrated DNA Technologies (IDT), Coralville, IA, USA), in a MultiTherm heat block (Benchmark Scientific, Sayreville, NJ, USA). Temperature (59–67 °C), reaction time (20–60 min), primer concentrations (five conditions) and twelve additives (Table 2) were optimised and evaluated on 2% agarose gels. The selected conditions were 65 °C for 40 min with 0.2 µM F3/B3, 1.6 µM FIP/BIP and 0.8 µM LF/LB (Figure 1). For detection, the LAMP product was mixed with 1× NEBuffer r2.1, 25 nM LbCas12a (New England Biolabs, Ipswich, MA, USA), 25 nM crRNA and 1.5 µM ssDNA FAM/quencher reporter, and fluorescence was read every 30 s at 42 °C for 25–30 min on the same real-time instruments used for qPCR. One microlitre of the LAMP product was added to the detection mix to give a final volume of 20 µL, and the ribonucleoprotein was not pre-incubated.

Reactions were classified as positive based solely on the endpoint fluorescence ratio (last/first reading ≥ 1.5). Time-to-positivity (TTP), defined as the interpolated time when baseline-corrected fluorescence (ΔR = R(t) − R at the first reading) first exceeded ten times the standard deviation of ΔR across negative controls in the same run (with a minimum threshold of 100 RFU), and T50, the time to reach half the maximum signal above the negative-control baseline, were calculated only for positive reactions. Two types of negative controls were included: a no-template control (water) and an *E. coli*-free whole-blood control (human DNA) processed through the complete workflow. The number of controls per run and the corresponding thresholds are reported in Table S8. The workflow required approximately 100 min from sample receipt to a positive result: 10 min for leukocyte depletion, 40 min for automated DNA extraction, 5 min for reaction setup and loading, 40 min for LAMP amplification, and 5 min for transfer and CRISPR/Cas12a detection. Negative results required an additional 25–30 min to be confirmed.

### 2.5. Analytical Specificity and Sequence Verification

Inclusivity and exclusivity were assessed on genomic DNA from 55 isolates (one reaction each), comprising clinical isolates from patients with BSI and reference strains (Table 3). The specificity panel comprised 15 Escherichia coli strains (13 clinical isolates, ATCC 25922, and K-12 MG1655) and 40 non-target isolates representing 17 species across 12 genera. The non-target panel included clinically relevant Gram-negative and Gram-positive bacteria, as well as *Candida albicans*, selected to reflect the aetiology of bloodstream infections at our centre, with emphasis on *Enterobacterales*. Human genomic DNA and no-template controls (NTCs) were also included. Detailed strain information is provided in Table 3 and Table S1. *Shigella* spp. and *Escherichia fergusonii*, which are closely related to *E. coli*, were not available and therefore could not be tested.

LAMP products were scored by agarose gel electrophoresis using the following criteria, adapted from the characteristic LAMP product patterns described by Notomi et al. [34]: +, ladder (more than three discrete bands with high-molecular-weight material at or near the well); ±, partial ladder or smear (three or fewer discrete bands, or continuous fluorescence without discrete bands); and −, no product. This scoring reflects product morphology rather than sequence identity, which was assessed by the CRISPR/Cas12a read-out. Electrophoresis was performed during assay development and on a subset of the specificity panel, but not in the limit-of-detection experiments or the clinical phase.

### 2.6. Limit of Detection

Twenty independently extracted replicates at 1, 5 and 10 CFU/mL, together with higher levels (10^2^, 10^3^ and 10^4^ CFU/mL; 2–6 replicates) and negative controls, were tested by both assays. The observed LOD was defined as the lowest concentration detected in ≥95% of replicates (≥19/20) [35]; this was 10 CFU/mL for both assays. Probit regression to all spike levels, *P* = Φ(*a* + *b*·log_10_*C*), and LOD*p* = 10^((Φ−1(p) − a)/b)^ [36]. Goodness of fit was assessed with Pearson’s χ^2^; when its significance was <0.15, a heterogeneity factor was applied [36]. 95% fiducial limits were calculated by Fieller’s method [37].

### 2.7. Conversion of CFU to dxs Copies

In exponential growth, the average copy number of a locus at relative position *m* be(SPSS PROBIT procedure, log_10_-transformed concentration) was fitted by maximum likelihood tween *oriC* (*m* = 0) and the terminus (*m* = 1) is *n* = 2^([*C*(1 − *m*) + *D*]/*τ*)^, where *C* (≈40 min) and *D* (≈20 min) are the replication and division periods and *τ* is the doubling time (Cooper-Helmstetter model) [38,39]. *dxs* lies 1.154 Mb from *oriC* (3,925,744 bp) on the 2.305 Mb clockwise replichore of the *E. coli* K-12 MG1655 genome (NC_000913.3) [40] (*m* = 0.50). At OD_600_ 0.400 in LB at 37 °C (*τ* ≈ 20–30 min), *n* = 2.5–4.0 (central value 3.0 at *τ* = 25 min); stationary cells carry one to two chromosomes [39]. One cell per CFU was assumed. As an empirical alternative, a single-hit Poisson model [41], *P* = 1 − exp(−*k* × 0.05 × *C*), was fitted to the detection data as a generalized linear model (binomial distribution, complementary log–log link, offset ln(0.05 × *C*)) [42] to estimate the number of amplifiable templates per nominal CFU (*k*) with Wald 95% CI; 95% detection requires ≥3 templates per reaction on average [33].

### 2.8. Clinical Samples and Blood Culture

Adults with clinically suspected BSI were enrolled prospectively at the 108 Military Central Hospital (Hanoi, Vietnam) between May 2022 and March 2023 (*n* = 52). Organ dysfunction was graded with the Sequential Organ Failure Assessment (SOFA) score according to Sepsis-3 [1]. Blood cultures were ordered by the treating physicians as part of routine clinical care and were processed independently of the study: bottles were incubated in a BD BACTEC 9120 system (Becton Dickinson, Franklin Lakes, NJ, USA) at 36 ± 0.5 °C for up to 5 days, and isolates from positive cultures were subcultured and identified by MALDI-TOF mass spectrometry (Bruker Daltonics, Bremen, Germany); culture results were retrieved from the hospital records. For molecular testing, a whole-blood sample was collected at a median of 0 days (IQR 0–1) after admission; the same sample was used for both qPCR and LAMP-CRISPR/Cas12a, so no additional blood was drawn for the index test. Patient Cq values were converted to bacterial load with the qPCR master curve and to dxs copies/mL with the growth-phase factor. Patients were classified as LAMP–CRISPR/Cas12a-positive if any reaction on their DNA eluate was positive. Two patients were tested in duplicate within a single run, and two were tested again in a later run where eluate remained; two approaches to interpreting replicate results were evaluated in sensitivity analyses. Pathogen names follow current nomenclature (*Enterobacter aerogenes* is reported as *Klebsiella aerogenes*).

### 2.9. Statistical Analysis

Continuous variables were tested for normality with the Shapiro-Wilk test [43]. Categorical variables are reported as *n* (%) and were compared with Fisher’s exact test [44]. Sensitivity, specificity, positive and negative percent agreement (PPA, NPA), inclusivity and exclusivity are given with 95% confidence intervals (CI) calculated by the Wilson score method. Agreement was assessed with Cohen’s *κ* (95% CI from the asymptotic standard error) and discordance with the exact McNemar test [45,46]. The reference standard for clinical accuracy was a composite of blood culture or qPCR detection of *E. coli*. Expected LAMP-CRISPR/Cas12a positives were computed by applying the analytical probit model to each patient’s Cq and compared with the observed number by the exact binomial test (one-tailed, as reported by SPSS when the test proportion differs from 0.5). TTP was compared with the Mann-Whitney U and Kruskal-Wallis tests [47], and correlations were assessed with Spearman’s ρ [48]. Unless stated otherwise, tests were two-sided with α = 0.05, using asymptotic significance for non-parametric tests; clinical comparisons were exploratory. Statistical analyses were performed in IBM SPSS Statistics, version 20 (IBM Corp., Armonk, NY, USA) and R, version 3.5.2 (https://www.r-project.org/). Figures were generated from the study data in Python (version 3.13) with matplotlib (version 3.11), using code drafted with the assistance of Claude Opus 5.5 (Anthropic, San Francisco, CA, USA); the tool was not used to generate, analyse or interpret the study data, nor to design the study. Reporting follows the MIQE [33], CLSI EP17 [35].

## 3. Results

### 3.1. Optimisation of LAMP and CRISPR/Cas12a Reactions

LAMP produced the characteristic ladder at all temperatures tested (59–67 °C) and reaction times of 30–60 min, and primer conditions with 1.2–1.6 µM FIP/BIP gave strong products without signal in the NTC (Figure 1A–C). The conditions carried forward were 65 °C for 40 min with primer condition B. For the Cas12a step, 25 nM crRNA gave a faster and higher fluorescence rise than 50 or 100 nM (Figure 1D).

**Figure 2:**
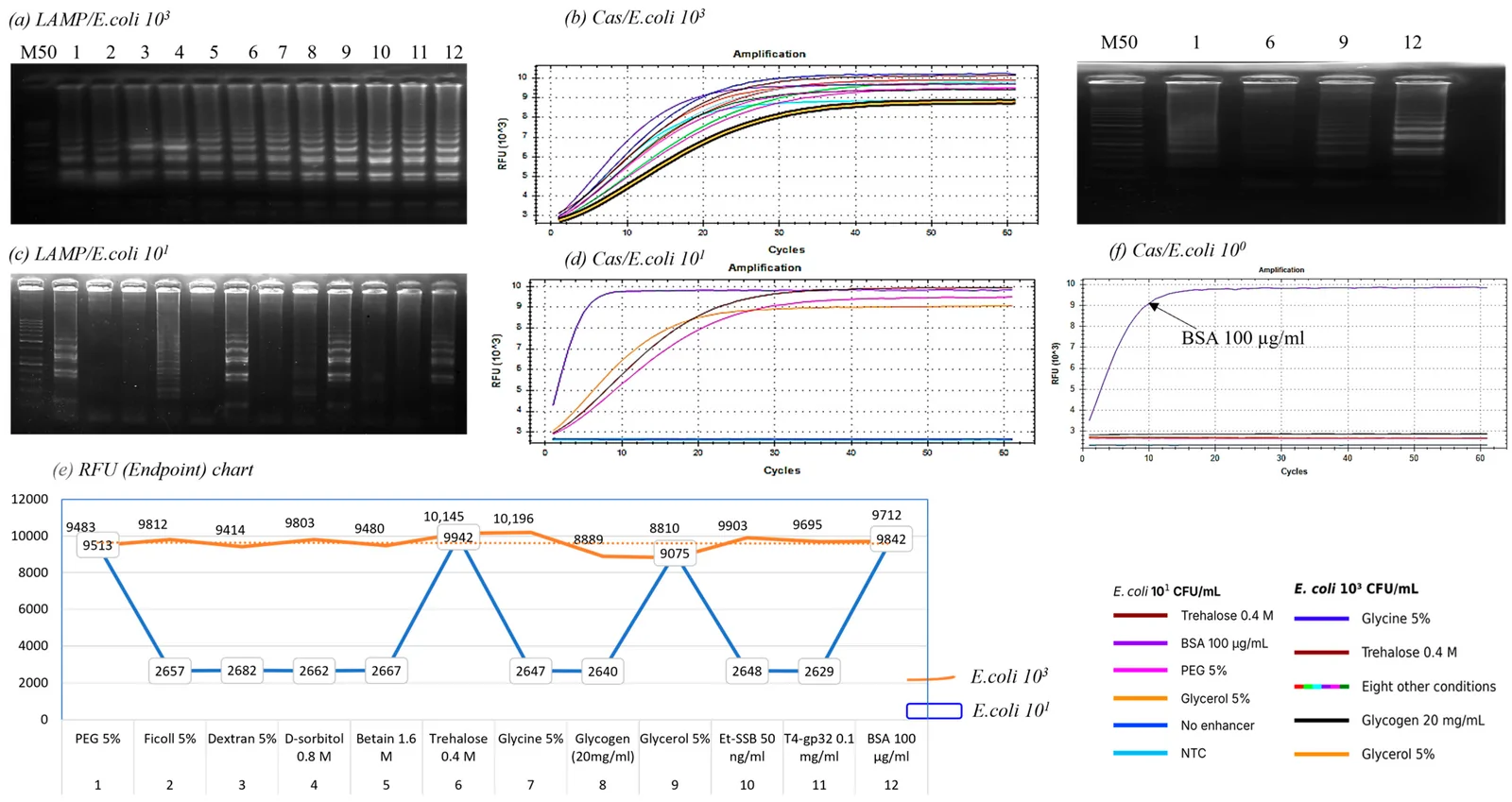
LAMP-CRISPR/Cas12a performance with different additives. (**a**,**b**) LAMP gel and Cas12a fluorescence at 10^3^ CFU/mL; (**c**,**d**) at 10^1^ CFU/mL; (**f**) Cas12a fluorescence at 10^0^ CFU/mL with BSA 100 µg/mL; (**e**) end-point fluorescence (RFU) at 10^3^ and 10^1^ CFU/mL for each additive (lane numbers as in Table 2).

The additive screen used one reaction per additive and per concentration and was exploratory: it was performed to rank the additives, not to measure analytical sensitivity. Because 10^0^ CFU/mL lies below the limit of detection established in Section 3.4, a positive or negative result at that concentration cannot be used to estimate the limit of detection. All twelve additives supported detection at 10^3^ CFU/mL, with tightly clustered end-point fluorescence (9611 ± 430 RFU). At 10^1^ CFU/mL, positive LAMP and Cas12a signals were obtained only with PEG 5%, trehalose 0.4 M, glycerol 5% or BSA 100 µg/mL, and at 10^0^ CFU/mL only BSA gave a positive Cas12a signal (Figure 2, Table 2). BSA concentrations were not titrated; the single concentration used was taken from the published report [49], in which BSA at this concentration protects enzyme activity in inhibitor-rich matrices. BSA was therefore retained in the final reaction, consistent with its known role in stabilising polymerase and Cas12a activity in complex samples.

### 3.2. Correspondence Between the LAMP and CRISPR/Cas12a Read-Outs on the Same Extracts

In a single run in which one set of extracts was carried through every read-out in parallel, shown in the Supplementary Materials as Figure S6 and from which no quantitative inference is drawn, qPCR (efficiency 91.3%, R^2^ = 0.998) and LAMP–CRISPR/Cas12a detected 10^1^–10^4^ CFU/mL, and qPCR additionally detected 10^0^ CFU/mL (Cq 39.46) in this single run (Figure S6). Stand-alone LAMP produced a ladder from the human-DNA negative control, whereas qPCR showed no amplification; the Cas12a step classified this sample as negative, in agreement with qPCR. The NTC was negative by all methods. In this single run the product obtained from the human DNA control was not reported by the Cas12a step, while the signal at and above 10 CFU/mL was retained; this is one observation and is not by itself evidence that non-specific signal is removed in general.

### 3.3. Analytical Specificity and Sequence Verification of the Target

**Figure 3:**
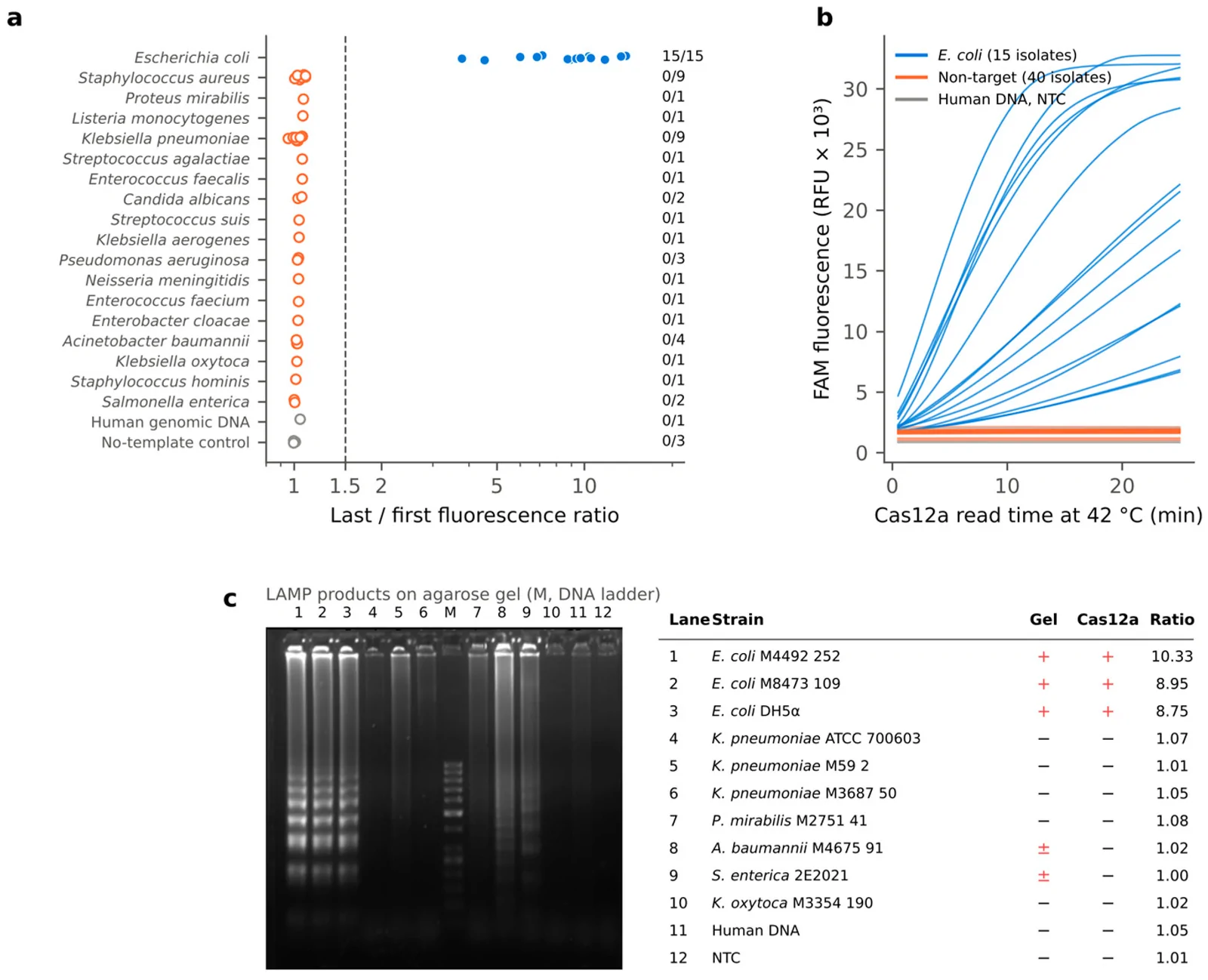
Analytical specificity of LAMP–CRISPR/Cas12a. (**a**) Last/first fluorescence ratio of 15 *E. coli* strains (blue, filled = positive) and 40 non-target isolates (orange, open = negative) by species, with human DNA and NTC (grey); dashed line, call threshold 1.5; numbers, positive/tested. (**b**) Raw Cas12a kinetics of the same reactions. (**c**) LAMP products on agarose gel (M, DNA ladder) with the gel score, the Cas12a call and the last/first fluorescence ratio for each lane. Gel scores: +, ladder (more than three discrete bands with high-molecular-weight material at or near the well); ±, partial ladder or smear (three or fewer discrete bands, or continuous fluorescence without discrete bands); −, no product. Cas12a calls: +, last/first fluorescence ratio ≥ 1.5; −, ratio below 1.5.

LAMP-CRISPR/Cas12a detected all 15 *E. coli* strains (inclusivity 100%, 95% CI 79.6–100) and none of the 40 non-target isolates (exclusivity 100%, 95% CI 91.2–100), human DNA or NTC (Table 3, Figure 3; per-isolate results in Table S1). Last/first fluorescence ratios were 3.78–13.85 for *E. coli* and 0.95–1.09 for non-target isolates. End-point fluorescence of *E. coli* (median 21,501 RFU, IQR 12,075–30,910) was well above that of non-target strains (median 1734 RFU, IQR 1663–1829; Mann–Whitney U, *p* < 0.001; Figure S1); the two *S. enterica* strains stayed at their initial reading (1170 and 1017 RFU; ratio 1.00 for both). On agarose gel, stand-alone LAMP produced a partial ladder or smear (±) for *A. baumannii* M4675 91 and *S. enterica* 2E2021, but both were negative after Cas12a (ratios 1.02 and 1.00; Figure 3c).

Sanger sequencing of the F3/B3 amplicon gave a clean single-peak trace of 208 bases, of which 207 bases of high quality were used as the BLASTn query (Figure S7, Table S2). The sequence matched *E. coli dxs* with 100% nucleotide identity over 99% of the query (bit score 369, E-value 1 × 10^−97^), and all top 100 BLASTn hits were *Escherichia coli* (*Enterobacteriaceae*). The amplified region therefore corresponds to the intended *dxs* target. BLASTn found no significant similarity between the dxs target region and *S. enterica* sequences, consistent with the absence of a Cas12a signal from the two *S. enterica* strains that produced a LAMP product.

Two closely related taxa were not represented in the panel. *Shigella* spp. match the amplicon at 100% identity, with every primer site and the protospacer conserved, and *Escherichia fergusonii* at 91.77–96.71%; neither was available to us as an isolate and cross-reaction with *Shigella* spp. is therefore predicted. The exclusivity figure applies to the panel as constituted, and the assay is described as detecting the *E. coli*/*Shigella* clade rather than *E. coli* sensu stricto (Supplementary Methods S1).

### 3.4. Limit of Detection in Whole Blood

**Figure 4:**
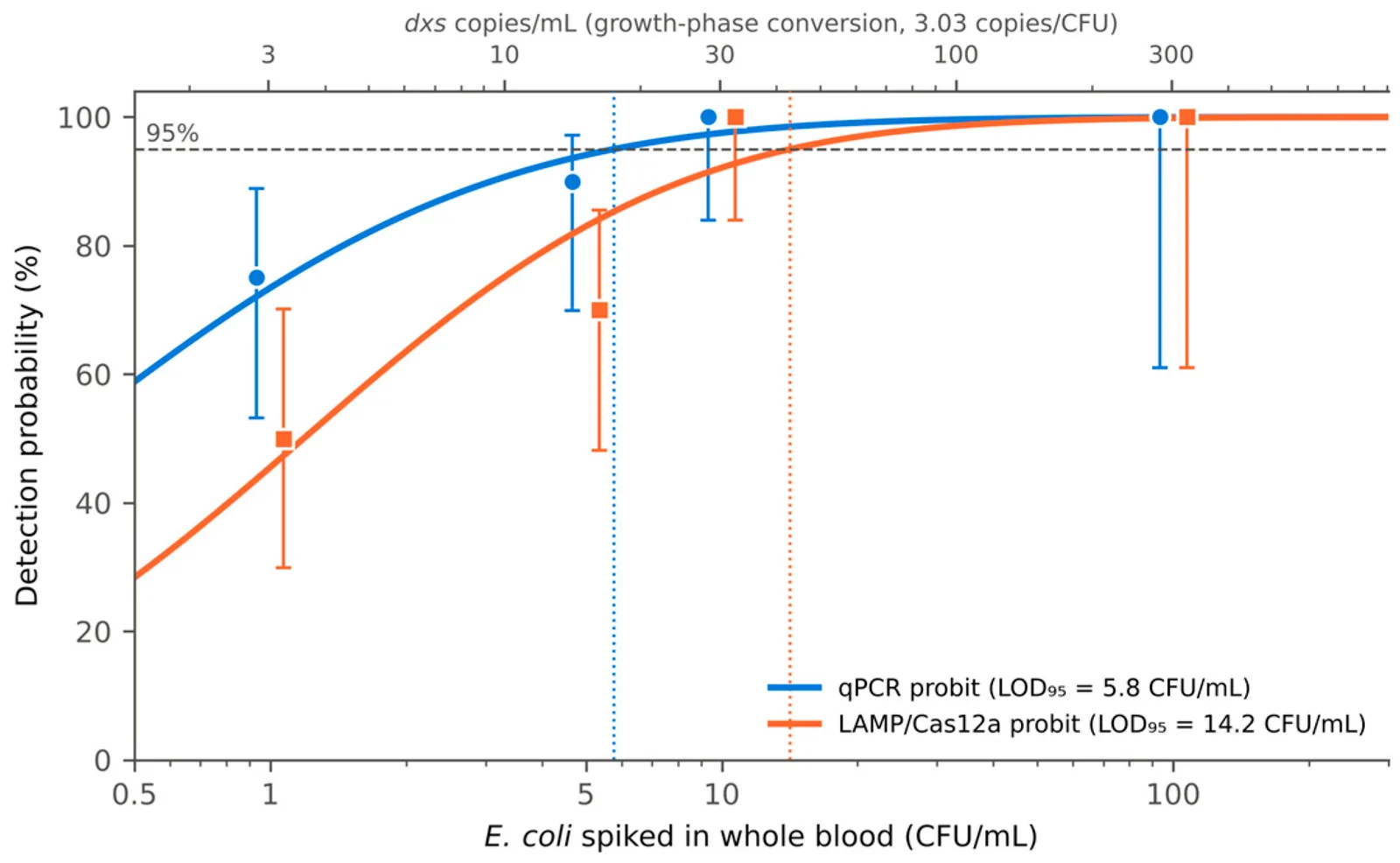
Probit detection curves for qPCR and LAMP–CRISPR/Cas12a in spiked whole blood. Lines, maximum-likelihood probit fits (SPSS PROBIT); points, observed detection rates with Wilson 95% CI. Dotted vertical lines mark LOD_95_: 5.8 CFU/mL (qPCR) and 14.2 CFU/mL (LAMP–CRISPR/Cas12a). Top axis, *dxs* copies/mL with the growth-phase factor (3.03 copies/CFU).

The qPCR standard curve was linear over 10^1^–10^4^ CFU/mL in six independent runs (R^2^ 0.995–0.9999); the master curve was Cq = −3.620·log_10_(CFU/mL) + 40.86 (R^2^ 0.9995, efficiency 88.9%; Figure S2, Table S3). Both assays detected 20/20 replicates at 10 CFU/mL, giving an observed LOD of 10 CFU/mL (0.5 CFU per reaction) for each. At 5 and 1 CFU/mL, qPCR detected 18/20 and 15/20 and LAMP-CRISPR/Cas12a 14/20 and 10/20 replicates (Table 4, Figure 4; Tables S4 and S5). LAMP-CRISPR/Cas12a positive and negative reactions were completely separated (ratios 2.69–8.59 vs. 0.99–1.03; Figure S3). Probit regression gave an LOD_95_ of 5.8 CFU/mL (95% fiducial limits 2.6–958) for qPCR and 14.2 CFU/mL (6.7–156) for LAMP-CRISPR/Cas12a, a 2.5-fold difference. Limits of detection are reported to one decimal place throughout; the unrounded probit estimates are 5.77 and 14.19 CFU/mL and the full set of detection probabilities is given in Table S5. Both slopes were significant (*p* = 0.018 and 0.001) and both models fitted the data (Pearson χ^2^ = 1.13 and 4.27, df = 4, *p* = 0.89 and 0.37), so no heterogeneity factor was applied. Upper fiducial limits were wide because only three concentrations lay within the 5–95% response range.

### 3.5. Conversion of the LOD to dxs Copies

**Figure 5:**
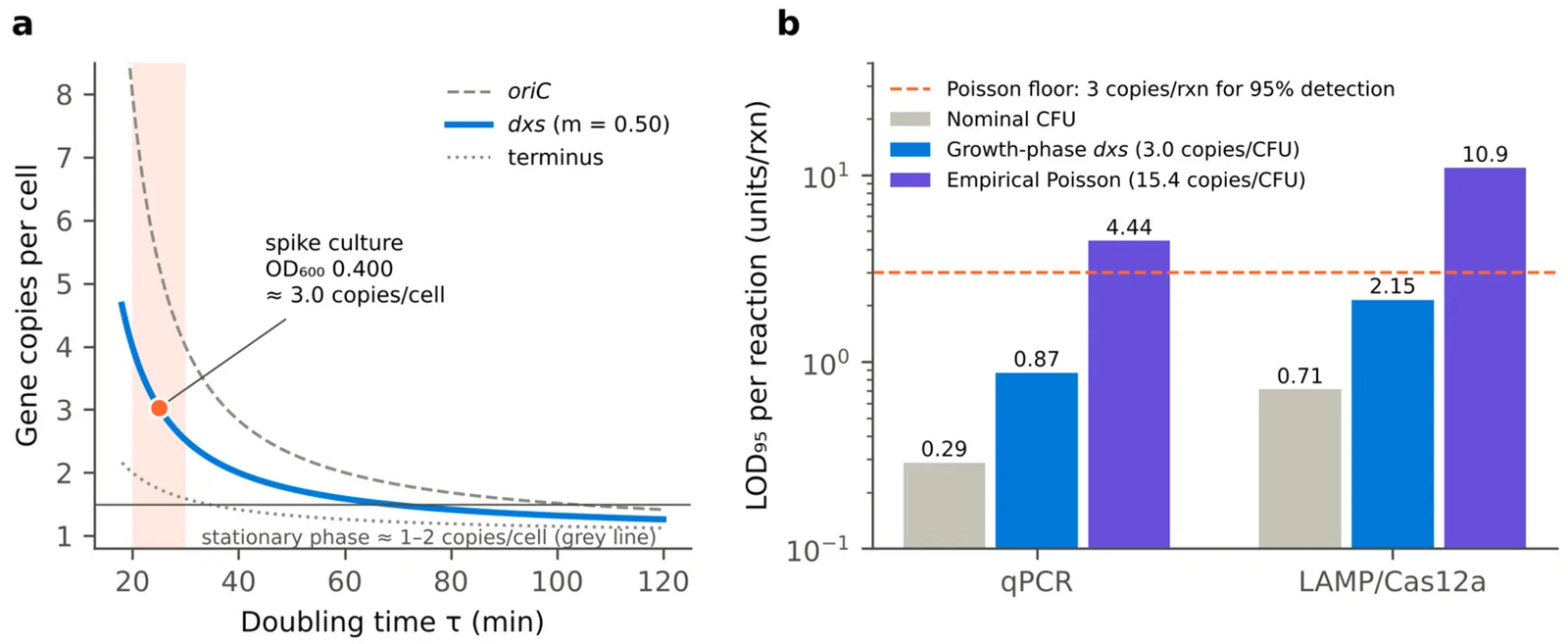
Conversion from CFU to *dxs* copies. (**a**) Gene copies per cell for *oriC*, *dxs* (*m* = 0.50) and the terminus as a function of doubling time (Cooper-Helmstetter model; *C* = 40 min, *D* = 20 min); the spike culture (OD_600_ 0.400, *τ* ≈ 25 min) carries ≈3.0 *dxs* copies per cell. (**b**) Probit LOD_95_ per reaction expressed as nominal CFU, growth-phase *dxs* copies and empirical Poisson templates; dashed line, Poisson floor of three templates per reaction for 95% detection.

The spike culture was harvested in mid-exponential phase, where the Cooper–Helmstetter model predicts 3.0 *dxs* copies per cell (2.5–4.0; Figure 5a). On this basis, the LOD_95_ corresponds to 17.5 *dxs* copies/mL for qPCR and 43.0 copies/mL for LAMP–CRISPR/Cas12a (Table 5; Table S6); with a stationary culture, the same CFU values would correspond to 6–12 and 14–28 copies/mL. This conversion, however, places both LOD_95_ values below the Poisson minimum of three templates per reaction (Figure 5b). The empirical single-hit model indicated 15.4 amplifiable templates per nominal CFU (95% CI 10.0–23.7), about five times the chromosomal estimate; with this factor, the qPCR LOD_95_ equals 4.4 templates per reaction, close to the theoretical floor.

### 3.6. Time-to-Positivity

**Figure 6:**
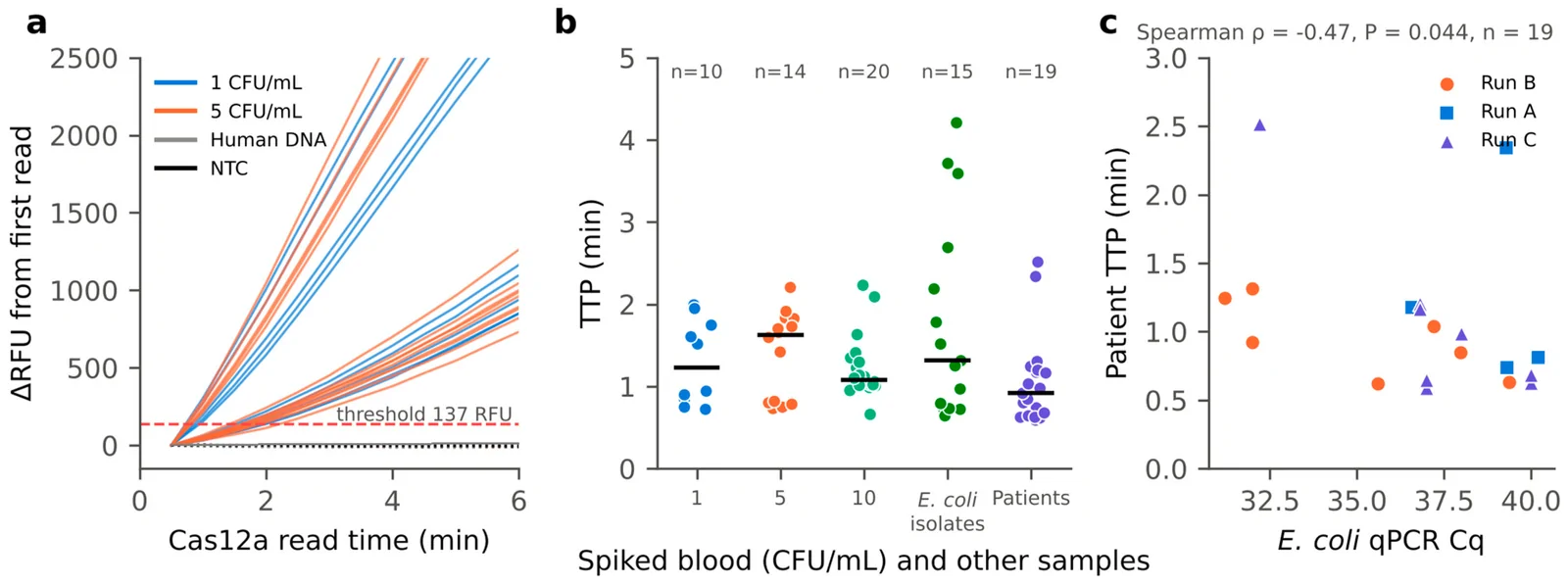
Time-to-positivity of LAMP–CRISPR/Cas12a. (**a**) Baseline-corrected fluorescence (ΔRFU from the first read) during the first 6 min of the LOD run; red dashed line, TTP threshold (137 RFU, 10 × SD of negative-control ΔR); dotted lines, negative controls. (**b**) TTP of positive reactions for spiked blood at 1, 5 and 10 CFU/mL, *E. coli* isolates of the specificity panel and patients (mean per patient); bars, medians. (**c**) Patient TTP versus *E. coli* qPCR Cq by run.

Positive LAMP-CRISPR/Cas12a reactions were read within minutes (Table 6, Figure 6; Tables S7 and S8). All 69 positive reactions in the LOD and patient runs crossed the TTP threshold within 4.3 min (median 1.1 min, IQR 0.84–1.68), as did all 15 *E. coli* isolates (median 1.3 min, maximum 4.2 min); no negative control or non-target reaction crossed it within 25–30 min. TTP did not differ between 1, 5 and 10 CFU/mL (Kruskal–Wallis *p* = 0.74), nor did T50 (*p* = 0.59). In patients, TTP was 0.9 min (IQR 0.6–1.2; n = 19) and was not positively related to load (Spearman ρ = −0.47 versus Cq, *p* = 0.044, computed on the combined data of the three runs (no samples were tested individually tested); T50 versus Cq, ρ = −0.24, *p* = 0.31).

### 3.7. Characteristics of the Clinical Cohort

The 52 patients had a median age of 66 years (IQR 58–80; mean 67.7 ± 16.0) and 34 (65.4%) were male (Table 7; per-patient data in Table S9). Forty-two (80.8%) met the Sepsis-3 criteria (SOFA ≥ 2), 8 (15.4%) had septic shock and in-hospital mortality was 30.8%. qPCR detected a pathogen in 47 patients (90.4%) and blood culture in 23 (44.2%). *E. coli* was the leading pathogen: 33 (63.5%) by qPCR, including one mixed detection with *Streptococcus* sp., and 17 (32.7%) by culture. Other qPCR detections were *K. pneumoniae* (*n* = 7), *Streptococcus* sp. (*n* = 2), *Enterococcus* sp. (*n* = 2), *A. baumannii*, *S. aureus* and *Streptococcus pneumoniae* (*n* = 1 each); other culture isolates were *K. pneumoniae* (*n* = 2), *S. aureus* (*n* = 2), *K. aerogenes* and *S. pneumoniae* (*n* = 1 each).

### 3.8. Diagnostic Performance of LAMP–CRISPR/Cas12a, qPCR and Blood Culture

**Figure 7:**
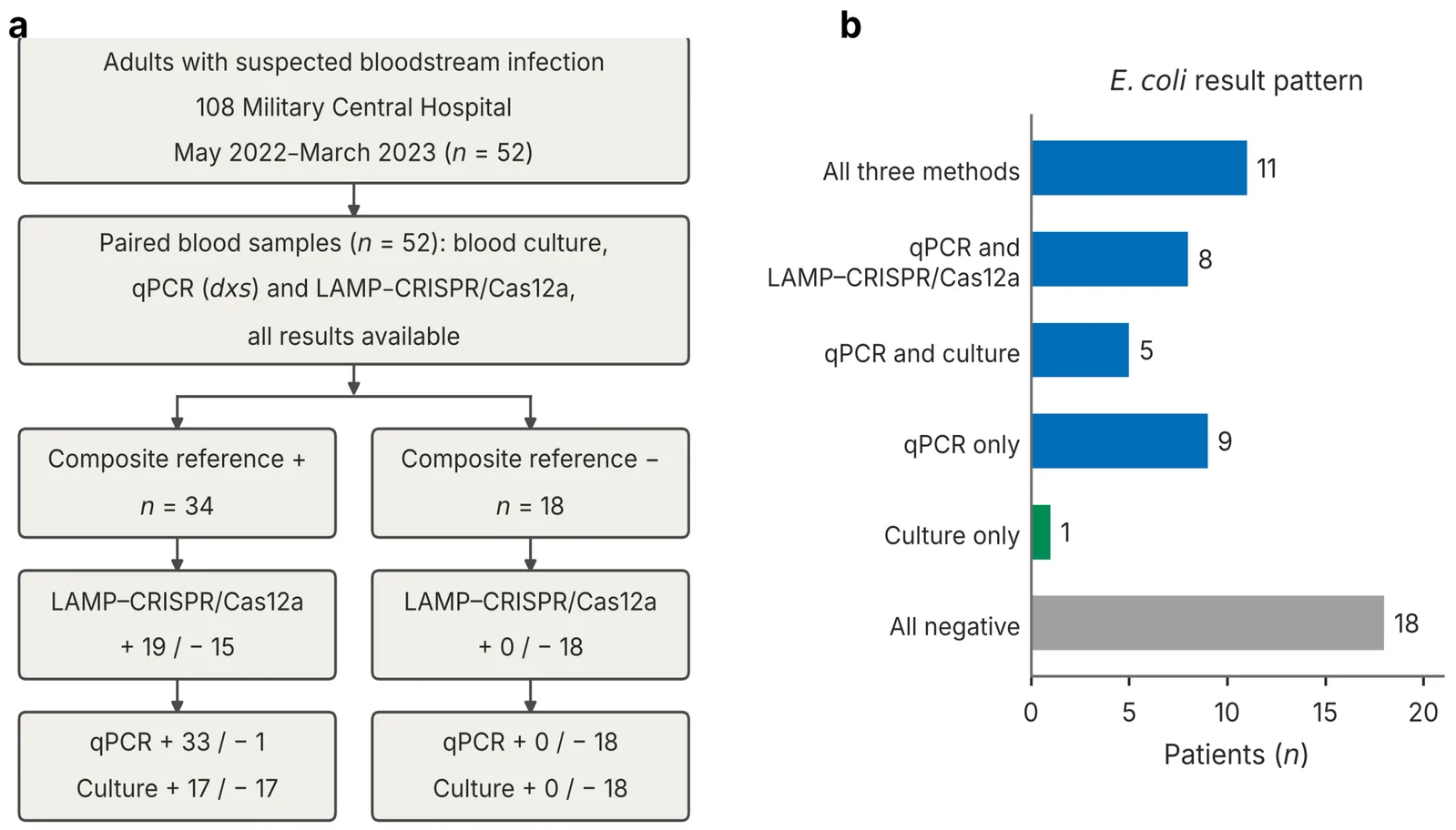
Patient flow and result patterns. (**a**) STARD-style flow diagram of the 52 patients against the composite reference (blood culture or qPCR *E. coli*). (**b**) Pattern of *E. coli* results across qPCR, LAMP-CRISPR/Cas12a and blood culture.

Against the composite reference (34 positive, 18 negative), sensitivity was 97.1% for qPCR, 55.9% for LAMP-CRISPR/Cas12a and 50.0% for blood culture, with 100% specificity for all three methods (Table 8, Figure 7; Table S10). Compared with qPCR, LAMP-CRISPR/Cas12a was positive in 19/33 qPCR-positive samples and negative in all 19 qPCR-negative samples (PPA 57.6%, 95% CI 40.8–72.8; NPA 100%; κ = 0.50). PPA decreased to 54.5% (95% CI 38.0–70.2) when between-run repeats were excluded and to 51.5% (95% CI 35.2–67.5) when all reactions were required to be positive. All discordant pairs were qPCR-positive and LAMP–CRISPR/Cas12a-negative (14 vs. 0; McNemar *p* < 0.001). qPCR detected 16 of 17 culture-confirmed E. coli infections and was also positive in 17 culture-negative cases, eight of which were independently confirmed by LAMP-CRISPR/Cas12a. Against blood culture, LAMP-CRISPR/Cas12a had a PPA of 64.7% and an NPA of 77.1%. The influence of the Cq cut-off was also assessed. Lowering the cut-off to 40.0 would classify three culture-confirmed patients with Cq values of 40.05, 40.20 and 40.30 as negative, while changing the probit LOD_95_ of the real-time PCR assay only from 5.77 to 5.86 CFU/mL. Raising the cut-off to 40.3 would exclude the single patient with Cq 40.50, who was culture-negative and LAMP-CRISPR/Cas12a-negative, increasing PPA from 57.6% to 59.4%. No study conclusion depended on the cut-off within this range.

### 3.9. Bacterial Load Explains Clinical Discordance

**Figure 8:**
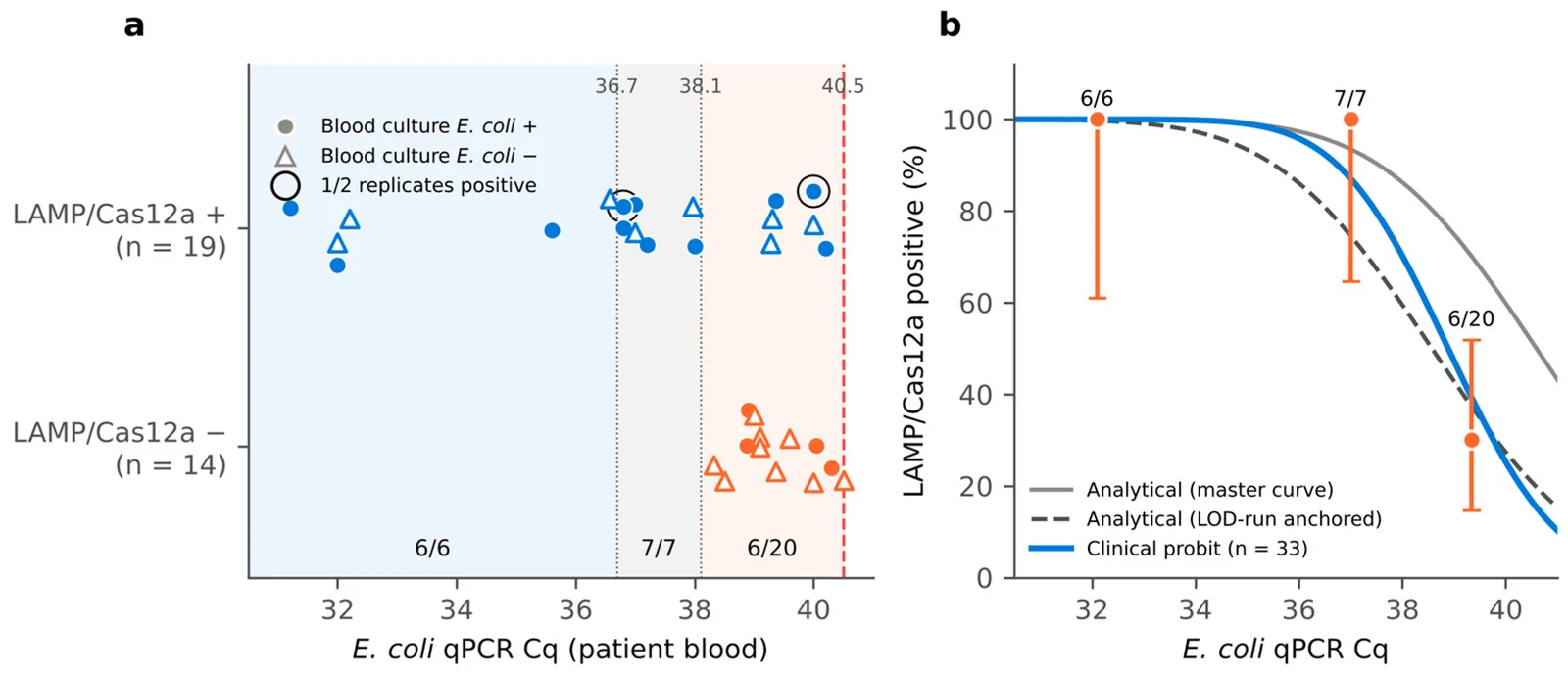
LAMP-CRISPR/Cas12a results in patients relative to *E. coli* load. (**a**) Cq of the 33 *E. coli* qPCR-positive patients by LAMP-CRISPR/Cas12a result; filled circles, culture-confirmed *E. coli*; open triangles, culture-negative; ringed, one of two replicate reactions positive. Shaded zones: at or above the LAMP-CRISPR/Cas12a LOD_95_ (Cq ≤ 36.7), between the two LOD_95_ values, and below the qPCR LOD_95_ (Cq > 38.1); red dashed line, Cq cut-off 40.5. (**b**) LAMP-CRISPR/Cas12a positivity versus Cq: analytical predictions through the master curve (grey) and with the LOD-run calibration (dashed), clinical probit fit (blue) and observed rates per zone with Wilson 95% CI.

*E. coli* loads in patient blood were very low. Cq values ranged from 31.2 to 40.5, corresponding to a median of 3.5 CFU/mL (IQR 2.4–12.4) or 10.5 *dxs* copies/mL (7.3–37.7). Twenty-seven of 33 samples (82%) had Cq > 36.7, i.e., below the LAMP–CRISPR/Cas12a LOD_95_, and 20 (61%) had Cq > 38.1, below the qPCR LOD_95_ itself. All 33 positive Cq values were ≤ 40.5; four were between 40.05 and 40.50, three of them culture-confirmed. LAMP-CRISPR/Cas12a detected 6/6 samples above its own LOD_95_, 7/7 between the two LOD_95_ values and 6/20 below the qPCR LOD_95_ (Table 9, Figure 8). The clinical probit curve reached 50% positivity at Cq 38.9 (95% fiducial limits 37.4–40.1). Applying the analytical probit model with the calibration of the LOD run predicted 17.9 positive patients, in agreement with the 19 observed (exact binomial test, one-tailed *p* = 0.42). LAMP-CRISPR/Cas12a-positive patients also had higher procalcitonin than LAMP-negative patients (median 72.2 vs. 6.3 ng/mL, *p* = 0.004), and lower Cq correlated with higher procalcitonin (ρ = −0.52, *p* = 0.004; Figure S4, Table S11).

## 4. Discussion

We developed a LAMP-CRISPR/Cas12a assay for *E. coli* that works on DNA extracted from 1 mL of whole blood, is fully specific on a 55-isolate panel and gives a positive read-out within 5 min of the Cas12a step. Its probit LOD_95_ was 14.2 CFU/mL, about 2.5-fold above that of qPCR (5.8 CFU/mL), while both assays detected all replicates at 10 CFU/mL. In patients with suspected BSI, the assay detected *E. coli* as often as blood culture (19 vs. 17 patients) without any false positives relative to qPCR.

The main advantage of the Cas12a step is specificity. Stand-alone LAMP generated products from human DNA and from two non-target strains (*A. baumannii* and *S. enterica*), which a turbidity or intercalating-dye read-out would have reported as positive. Cas12a *trans*-cleavage requires base pairing between the crRNA and the amplicon next to a TTTN motif [18], and none of these products activated it. Sanger sequencing confirmed that the amplified region is the intended *E. coli dxs* sequence (100% identity), so positive Cas12a signals report the target itself rather than an off-target product. Specificity is particularly important in blood, where human DNA exceeds bacterial DNA by several orders of magnitude [12,13], and in *Enterobacterales*, where *Salmonella*, *Klebsiella* and *Enterobacter* share conserved housekeeping genes with *E. coli*.

The clinical sensitivity of LAMP-CRISPR/Cas12a (57.6% against qPCR) reflects the bacterial load of the patients rather than a failure of the assay. Most *E. coli* bacteraemias in this cohort carried fewer than 6 CFU/mL, consistent with previous reports [11], and 61% of qPCR-positive samples were below the qPCR LOD_95_ itself. LAMP-CRISPR/Cas12a detected every sample at or above the qPCR LOD_95_, and the number of positives predicted from the spiked-blood probit model matched the number observed once the qPCR calibration of the LOD run was used. The remaining difference with the averaged six-run standard curve corresponds to a 2-Cq calibration offset and underlines the value of run-specific calibrators when Cq values are converted to load.

Finally, the replicate discordance seen in four patients is a direct consequence of operating at the limit of detection rather than a defect of the calling rule. The estimated loads of those patients were 1.7, 3.5, 11.6 and 13.2 CFU/mL, all at or below the LOD_95_, which is precisely the range in which a reaction succeeds or fails according to whether a template is sampled. Section 3.8 reports the positive percent agreement under three calling rules, 57.6%, 54.5% and 51.5%, which differ by at most six percentage points.

The 17 qPCR-positive, culture-negative samples had the same Cq distribution as culture-confirmed cases, and LAMP-CRISPR/Cas12a, which uses different primers and a different read-out, confirmed eight of them. This suggests that most represent true low-level bacteraemia missed by culture, a known consequence of prior antibiotic exposure (30% in this cohort) and small sampled volumes [7]. LAMP-CRISPR/Cas12a positivity was associated with higher load and procalcitonin, so the assay preferentially detected patients with a larger bacterial burden and stronger host response.

Expressing LOD in CFU/mL is common but depends on the culture. Because *dxs* lies at mid-replichore, a mid-exponential cell carries about three copies, compared with one or two in stationary phase. Stating the growth phase therefore matters: the limit of detection of 14.2 CFU/mL corresponds to ≈ 43 copies/mL for the mid-exponential culture used here but to only 14–28 copies/mL for a stationary culture, and the corresponding figures for the real-time PCR assay are 17.5 and 5.8–11.5 *dxs* copies/mL depending on the culture. Our data also show that nominal CFU underestimate the number of amplifiable templates: the observed detection rates require about 15 templates per CFU, likely because of extracellular DNA and non-culturable cells in the spike, cell aggregates counted as single colonies and uncertainty in the OD-to-CFU conversion. A digital PCR-quantified genomic DNA standard spiked in parallel would allow a copy-based LOD to be reported directly, as recommended by the MIQE guidelines [33].

Comparison with recent LAMP-CRISPR/Cas12a and digital-PCR-based assays. Our choice of whole blood rather than plasma as the input matrix is supported by the only prospective study to have compared the two directly for molecular pathogen detection. In 223 intensive-care patients with clinically suspected sepsis, of whom 62 (27.8%) had positive blood cultures, paired plasma and whole-blood multiplex droplet digital PCR performed similarly overall, but whole blood gave a markedly higher detection rate for the *Candida* genus (87.5% versus 31.3%, *p* = 0.03), and both matrices provided microbiological support for a substantial number of culture-negative patients adjudicated as definite sepsis (38 and 46 cases respectively) [47]. That study attributes the advantage of whole blood to a pretreatment that lyses erythrocytes and disrupts cellular components before extraction, thereby recovering organisms retained in the cellular fraction that cell-free plasma assays miss. The MCLB-1 leukocyte-depletion step used here follows the same principle, and our finding that 17 of 52 patients were *E. coli* qPCR-positive but culture-negative is consistent with their observation that molecular testing of whole blood recovers infections that culture does not.

Where our assay sits on analytical sensitivity depends heavily on the matrix and on whether the sample is concentrated. A one-pot LAMP-CRISPR/Cas12b assay for *Klebsiella pneumoniae* reported a limit of detection of 1 pg of genomic DNA, equivalent to 160 cells per reaction or 1.6 × 10^5^ CFU/mL of sample, with 100% sensitivity and 96% specificity on 58 sputum specimens [48]; our LOD_95_ of 14.2 CFU/mL of whole blood is four orders of magnitude lower, but the comparison is not one of chemistry. The difference is almost entirely attributable to sample preparation, since 1 mL of blood is depleted of host cells and concentrated into a 100 µL eluate before amplification. At the other extreme, a target-amplification-free CRISPR-CasΦ assay with a collateral-cleavage-enhancing amplifier reached 0.11 copies/µL and detected bacteria at 1.2 CFU/mL in serum within 40 min [49], about tenfold below our figure without any pre-amplification at all. We therefore do not present this assay as the most sensitive of its class; its contribution is the combination of an enrichment step suited to whole blood with a sequence-verified read-out, evaluated prospectively against both culture and real-time PCR.

These comparisons also indicate where the two-step format should go next. Both of the assays above are performed in a single closed tube, which removes the principal route of amplicon carry-over. One obstacle to converting our assay is that the protospacer must lie between the F1 and B1 sites and be preceded by a protospacer-adjacent motif, which left only two candidate positions in the *dxs* stem; the *K. pneumoniae* assay circumvents this by engineering the motif into the forward inner primer, so that a suitable natural sequence is no longer required [48]. Adopting that approach, together with the enrichment step already validated here, is the most direct route to a one-pot *E. coli* assay for whole blood.

The Cas12a read-out was fast: every positive crossed the TTP threshold within 4.3 min, so the detection step could be shortened from 20 min to 5 min. TTP did not vary with input, because Cas12a was read after LAMP had reached its end-point; it should be regarded as a speed metric rather than a quantitative one. A one-pot, real-time format would be needed for quantitative TTP.

This study has several limitations. Sample processing, including leukocyte depletion and magnetic-bead extraction, requires laboratory equipment, and simpler extraction methods with lateral-flow or portable fluorescence readouts are needed for point-of-care applications. The specificity assessment was limited by the use of a single reaction per isolate, non-normalised DNA inputs, and the omission of closely related species, including *Shigella* spp. and *Escherichia fergusonii*. The clinical cohort was small and single-centre, and the inclusion of qPCR in the composite reference standard may have inflated its apparent accuracy. Furthermore, LAMP-CRISPR/Cas12a was evaluated on stored DNA after qPCR in a subset of patients, with discordant replicate results observed in two cases. Importantly, the positive percent agreement with real-time PCR was only 57.6%, indicating that the assay is not suitable as a stand-alone rule-out test for E. coli bacteraemia and should instead be considered a rapid confirmatory method alongside blood culture. The two-step workflow also requires opening tubes containing high concentrations of LAMP amplicons, creating a risk of carryover contamination despite the use of physically separated work areas, a unidirectional workflow, and consistently negative no-template controls. A closed-tube or lateral-flow format would help address this issue. Analytical sensitivity may also be limited because only 5% of each eluate was tested, while the wide confidence limits around the estimated limits of detection reflect uncertainty in the available spike-level data. In addition, the contribution of BSA was not optimised through concentration titration. Finally, three of six qPCR standard curves had efficiencies below the 90% threshold recommended by MIQE [33], with residual inhibition across extraction batches a plausible explanation for the observed variation. Consequently, back-calculated bacterial loads should be regarded as approximate estimates rather than precise quantitative measurements. However, because this study primarily evaluated the qualitative detection performance of LAMP-CRISPR/Cas12a relative to real-time PCR and blood culture, rather than absolute bacterial quantification, this limitation does not directly affect the main performance assessment, which relied on predefined classification cut-offs. Run-specific calibrators are nevertheless advisable when converting Cq values into quantitative estimates.

## 5. Conclusions

LAMP-CRISPR/Cas12a detects *E. coli* in whole blood with no cross-reaction among the 40 non-target isolates tested, an LOD_95_ of 14.2 CFU/mL (≈43 *dxs* copies/mL for a mid-exponential culture) and a read-out within 5 min, and the Cas12a step did not report the non-specific LAMP products observed in this study. In patients with suspected BSI, it detected *E. coli* as often as blood culture with no false positives relative to qPCR, but its sensitivity is limited by the very low bacterial loads of bacteraemia. The assay is therefore suitable as a specific confirmatory test rather than a stand-alone rule-out test. Improved enrichment of bacterial DNA from larger blood volumes and validation in larger, multicentre cohorts are the next steps.

## Figures and Tables

**Table 1:** LAMP primers, crRNA, reporter and real-time PCR oligonucleotides targeting the *E. coli dxs* gene.

Name	Sequence (5′ → 3′)	Position (NC_000913.3)	Source
F3-Dxs	GTCGATGGCGAACAGGACC	438,921–438,939	This study
B3-Dxs	CTGATGGCGATTACTCCGG	439,145–439,163	This study
FIP-Dxs	CCATTGTCGCGATTTACT-CCACGTCATGCAGCACCTGAT	F1c 439,001–439,018; F2 438,956–438,976	This study
BIP-Dxs	GTCACCGCGTGTTGCTCGG-GTGAAGGTTCCGGCATGGTC	B1c 439,054–439,072; B2 439,119–439,138	This study
LF-Dxs	CTTTCCTGCAACGCGCCTAT	438,978–438,997	This study
LB-Dxs	TAGCGATCCGGGAATTTACGTGA	439,090–439,112	This study
DXS-crRNA	UAAUUUCUACUAAGUGUAGAU-CCGCCGGUCUGGCGAUUGGU	spacer 439,029–439,048	This study
ssDNA reporter (FQ)	/56-FAM/TTATT/3IABkFQ/	–	[32]
Dxs-F (qPCR)	GGCGAGAAACTGGCGATCCTTA	–	[30]
Dxs-R (qPCR)	CTTCATCAAGCGGTTTCACA	–	[30]
Dxs-probe (qPCR)	FAM-TTTGGTACGCTGATGCCAGACG-BHQ1	–	[31]

FIP-Dxs is F1c + F2 and BIP-Dxs is B1c + B2, the two components of each inner primer being separated by a hyphen; the crRNA comprises a 21-nt LbCas12a direct repeat followed by the 20-nt spacer, and only the spacer maps to the genome. The protospacer lies between the F1 and B1 sites and is preceded by a TTTG protospacer-adjacent motif at 439,049–439,052. The outer amplicon is 243 bp (438,921–439,163). Coordinates are given on NC_000913.3.

**Table 2:** Effect of additives on LAMP and LAMP–CRISPR/Cas12a detection of *E. coli*.

No.	Additive	10^3^ CFU/mL LAMP	10^3^ Cas12a	10^1^ CFU/mL LAMP	10^1^ Cas12a	10^0^ CFU/mL LAMP	10^0^ Cas12a
1	PEG 5%	+	+	+	+	±	−
2	Ficoll 5%	+	+	−	NA	NA	NA
3	Dextran 5%	+	+	−	NA	NA	NA
4	D-sorbitol 0.8 M	+	+	±	NA	NA	NA
5	Betaine 1.6 M	+	+	−	NA	NA	NA
6	Trehalose 0.4 M	+	+	+	+	±	−
7	Glycine 5%	+	+	−	NA	NA	NA
8	Glycogen 20 mg/mL	+	+	−	NA	NA	NA
9	Glycerol 5%	+	+	+	+	±	−
10	ET-SSB 50 ng/mL	+	+	−	NA	NA	NA
11	T4 gp32 0.1 mg/mL	+	+	−	NA	NA	NA
12	BSA 100 µg/mL	+	+	+	+	+	+

+, ladder (more than three discrete bands with high-molecular-weight material at or near the well); ±, partial ladder or smear (three or fewer discrete bands, or continuous fluorescence without discrete bands); −, no product; NA, not tested. BSA, 100 µg/mL per reaction.

**Table 3:** Inclusivity and exclusivity of LAMP–CRISPR/Cas12a (one reaction per isolate; positive, last/first fluorescence ratio ≥ 1.5).

Organism	Isolates (Reference Strains)	Positive	Ratio, Range
*Escherichia coli*	15 (ATCC 25922, DH5α)	15	3.78–13.85
*Staphylococcus aureus*	9 (ATCC 29213)	0	1.00–1.09
*Klebsiella pneumoniae*	9 (ATCC 700603)	0	0.95–1.07
*Acinetobacter baumannii*	4	0	1.02
*Pseudomonas aeruginosa*	3 (ATCC 27853)	0	1.03–1.04
*Candida albicans*	2 (ATCC 14053)	0	1.03–1.06
*Salmonella enterica*	2	0	1.00
Other species ^a^	11 (1 each)	0	1.02–1.08
Human genomic DNA	1	0	1.05
No-template control	3	0	1.00–1.01
Non-target total	40 (17 species, 12 genera)	0 (95% CI 0–8.8%)	0.95–1.09

^a^ *Enterobacter cloacae*, *Enterococcus faecalis*, *Enterococcus faecium*, *Klebsiella aerogenes*, *Klebsiella oxytoca*, *Listeria monocytogenes*, *Neisseria meningitidis* serogroup B, *Proteus mirabilis*, *Staphylococcus hominis*, *Streptococcus agalactiae* and *Streptococcus suis*.

**Table 4:** Detection rates and limits of detection in spiked whole blood (1 CFU/mL = 0.05 CFU per reaction). Four negative reactions were run for the real-time PCR assay: within the limit-of-detection run, a human DNA control, that is *E. coli*-free whole blood carried through MCLB-1 depletion and SaMag extraction exactly as a specimen, together with a no-template control of water; and a further pair of the same two kinds within the standard-curve runs. None produced a Cq. The Cq and end-point ΔR of all 62 reactions are given in Data S1.

Parameter	qPCR	LAMP–CRISPR/Cas12a
10^4^/10^3^/10^2^ CFU/mL, n/N	2/2 · 2/2 · 6/6	2/2 · 2/2 · 6/6
10 CFU/mL, *n*/N (%; 95% CI)	20/20 (100; 83.9–100)	20/20 (100; 83.9–100)
5 CFU/mL, *n*/N (%; 95% CI)	18/20 (90; 69.9–97.2)	14/20 (70; 48.1–85.5)
1 CFU/mL, *n*/N (%; 95% CI)	15/20 (75; 53.1–88.8)	10/20 (50; 29.9–70.1)
Negative controls	0/4	0/9
Observed LOD (≥95%)	10 CFU/mL	10 CFU/mL
Probit slope (SE); *p*	1.34 (0.57); 0.018	1.53 (0.45); 0.001
Pearson χ^2^ (df = 4); *p*	1.13; 0.89	4.27; 0.37
LOD_50_, CFU/mL (95% fiducial limits)	0.34 (<0.01–0.95)	1.19 (0.29–2.12)
LOD_95_, CFU/mL (95% fiducial limits)	5.8 (2.6–958)	14.2 (6.7–156)
LOD_99_, CFU/mL (95% fiducial limits)	18.6 (5.9–7.6 × 10^5^)	39.7 (13.5–1685)
LOD_95_, CFU per reaction	0.29	0.71
LOD_95_, *dxs* copies/mL ^a^	17.5 [89]	43.0 [218]
Cq at LOD_95_ (master curve)	38.1	36.7

^a^ Growth-phase factor 3.03 copies/CFU; in brackets, empirical factor 15.4 templates/CFU (Section 3.5).

**Table 5:** Conversion of the probit LOD_95_ from CFU to *dxs* copies according to growth phase of the spike culture (*dxs* at m = 0.50; C = 40 min, D = 20 min).

Growth Phase	*τ* (Min)	*dxs* Copies/CFU	qPCR LOD_95_, Copies/mL (per Reaction)	LAMP–CRISPR/Cas12a LOD_95_, Copies/mL (per Reaction)
Fast exponential	20	4.00	23.1 (1.15)	56.7 (2.83)
Mid-exponential, OD_600_ 0.400 (this study)	25	3.03	17.5 (0.87)	43.0 (2.15)
Late exponential	40	2.00	11.5 (0.58)	28.4 (1.42)
Slow growth	60	1.59	9.2 (0.46)	22.5 (1.13)
Stationary	–	1–2	5.8–11.5 (0.29–0.58)	14.2–28.4 (0.71–1.42)
Empirical Poisson (data-derived)	–	15.4 (10.0–23.7)	89 (4.4)	218 (10.9)

**Table 6:** Time-to-positivity (TTP) and time to half-maximal signal (T50) of LAMP–CRISPR/Cas12a.

Sample	Positive/Tested	TTP, Min, Median (IQR)	TTP Range, Min	T50, Min, Median (IQR)
Spiked blood, 1 CFU/mL	10/20	1.2 (0.8–1.8)	0.7–2.0	9.2 (5.4–13.8)
Spiked blood, 5 CFU/mL	14/20	1.6 (0.8–1.8)	0.7–2.2	12.9 (5.7–13.8)
Spiked blood, 10 CFU/mL	20/20	1.1 (1.0–1.3)	0.7–2.2	9.3 (8.6–11.1)
Positive control	3/3	1.8 (0.7–2.1)	0.7–2.1	13.9 (4.3–15.0)
*E. coli* isolates	15/15	1.3 (0.7–2.7)	0.6–4.2	13.0 (7.1–14.0)
Patients (one value per patient)	19/33 ^a^	0.9 (0.6–1.2)	0.6–2.5	7.4 (5.9–10.9)
Non-target isolates and negative controls	0/59	Not reached in 25–30 min	–	–

^a^ *E. coli* qPCR-positive patients. Thresholds (10 × SD of negative-control ΔR) were 100–368 RFU depending on run.

**Table 7:** Clinical and laboratory characteristics of patients with suspected bloodstream infection, by *E. coli* qPCR result.

Characteristic	All (*n* = 52)	*E. coli* qPCR+ (*n* = 33)	*E. coli* qPCR− (*n* = 19)	*p*
Age, years	66.0 (58.0–80.2)	69.0 (60.5–81.0)	62.0 (54.0–78.0)	0.183
Male sex	34 (65.4)	20 (60.6)	14 (73.7)	0.382
Diabetes mellitus	20 (38.5)	14 (42.4)	6 (31.6)	0.558
Hypertension	29 (55.8)	19 (57.6)	10 (52.6)	0.778
Chronic liver disease	5 (9.6)	5 (15.2)	0 (0.0)	0.145
Source: urinary tract	13 (25.0)	9 (27.3)	4 (21.1)	0.746
Source: abdominal	14 (26.9)	10 (30.3)	4 (21.1)	0.534
Source: lung	16 (30.8)	8 (24.2)	8 (42.1)	0.220
Source: unknown	13 (25.0)	8 (24.2)	5 (26.3)	1.000
SOFA score	3.5 (2.0–5.8)	4.0 (2.0–6.0)	2.0 (1.0–5.0)	0.078
SOFA ≥ 2	42 (80.8)	28 (84.8)	14 (73.7)	0.467
Septic shock	8 (15.4)	7 (21.2)	1 (5.3)	0.232
Mechanical ventilation	9 (17.3)	6 (18.2)	3 (15.8)	1.000
Continuous renal replacement therapy	6 (11.5)	5 (15.2)	1 (5.3)	0.397
White blood cells, ×10^9^/L	13.5 (9.5–20.0)	14.3 (9.5–19.6)	11.4 (9.3–21.8)	0.857
Platelets, ×10^9^/L	161 (110–222)	153 (83–215)	201 (142–267)	0.102
Procalcitonin, ng/mL ^a^	17.0 (1.7–81.7)	24.2 (4.4–100.0)	4.4 (1.1–32.7)	0.052
Antibiotics before sampling ^a^	14 (30.4)	9 (30.0)	5 (31.2)	1.000
Blood culture positive, any	23 (44.2)	17 (51.5)	6 (31.6)	0.247
Blood culture *E. coli*	17 (32.7)	16 (48.5)	1 (5.3)	0.002
LAMP–CRISPR/Cas12a positive	19 (36.5)	19 (57.6)	0 (0.0)	<0.001
In-hospital death	16 (30.8)	11 (33.3)	5 (26.3)	0.758
Length of stay, days	15.0 (10.0–23.0)	14.0 (8.5–19.5)	18.0 (11.0–30.0)	0.106

Data are *n* (%) or median (IQR); Fisher’s exact or Mann–Whitney *U* test. ^a^ Missing: procalcitonin 8, antibiotics 6.

**Table 8:** Diagnostic performance for *E. coli* bloodstream infection (*n* = 52).

Index Test	Reference	TP/FN/FP/TN	Sensitivity or PPA, % (95% CI)	Specificity or NPA, % (95% CI)	κ (95% CI)
qPCR	Composite ^a^	33/1/0/18	97.1 (85.1–99.5)	100 (82.4–100)	0.96 (0.88–1.00)
LAMP-CRISPR/Cas12a	Composite ^a^	19/15/0/18	55.9 (39.5–71.1)	100 (82.4–100)	0.47 (0.27–0.66)
Blood culture	Composite ^a^	17/17/0/18	50.0 (34.1–65.9)	100 (82.4–100)	0.41 (0.22–0.60)
LAMP-CRISPR/Cas12a	qPCR	19/14/0/19	57.6 (40.8–72.8) ᵇ	100 (83.2–100) ᵇ	0.50 (0.30–0.69)
qPCR	Blood culture	16/1/17/18	94.1 (73.0–99.0)	51.4 (35.6–67.0)	0.37 (0.17–0.56)
LAMP-CRISPR/Cas12a	Blood culture	11/6/8/27	64.7 (41.3–82.7) ᵇ	77.1 (61.0–87.9) ᵇ	0.41 (0.15–0.67)

Wilson 95% CI. ^a^ *E. coli* detected by blood culture or qPCR. ᵇ Positive/negative percent agreement.

**Table 9:** LAMP–CRISPR/Cas12a positivity among *E. coli* qPCR-positive patients by Cq zone.

Cq Zone	Load, CFU/mL (*dxs* Copies/mL)	*n*	LAMP–CRISPR/Cas12a+, *n* (%; 95% CI)	Expected ^a^	Blood Culture *E. coli*+
≤36.7 (≥LAMP LOD_95_)	≥14.2 (≥43)	6	6 (100; 61.0–100)	5.7	3
36.7–38.1 (between LOD_95_ values)	5.8–14.2 (17–43)	7	7 (100; 64.6–100)	5.0	5
38.1–40.5 (<qPCR LOD_95_)	<5.8 (<17)	20	6 (30; 14.5–51.9)	7.2	8
All	–	33	19 (57.6; 40.8–72.8)	17.9	16

^a^ Expected number of positives from the analytical probit model, with the qPCR calibration of the LOD run (10 CFU/mL at Cq 35.2).

## Data Availability

Reaction-level LAMP–CRISPR/Cas12a data, the specificity panel results, the de-identified clinical dataset and the SPSS syntax supporting the conclusions of this article are provided in the Supplementary Materials (Tables S1–S11, Data S1). Further data are available from the corresponding author on reasonable request.

## Supplementary Materials

The following supporting information can be downloaded at: https://www.mdpi.com/article/10.3390/diagnostics16213692/s1, Supplementary Methods S1: Design of the LAMP primers and the crRNA and their in silico validation [18,50,51,52]; Figure S1: End-point Cas12a fluorescence of the specificity panel; Figure S2: qPCR standard curves; Figure S3: LAMP-CRISPR/Cas12a raw kinetic data in the LOD experiments; Figure S4: *E. coli* qPCR Cq versus procalcitonin and platelet count; Table S1: Specificity panel results for each isolate; Table S2: Sequence verification of the *dxs* target; Table S3: qPCR standard-curve parameters by run; Table S4: Detection rates in spiked whole blood; Table S5: Probit analysis with 95% fiducial limits; Table S6: Conversion of CFU to *dxs* copies by growth phase; Table S7: Reaction-level LAMP–CRISPR/Cas12a results; Table S8: TTP and T50 by experiment and sample group; Table S9: De-identified per-patient results; Table S10: Agreement between methods; Table S11: Characteristics of *E. coli* qPCR-positive patients by LAMP-CRISPR/Cas12a and blood-culture result; Figure S5: Spiking scheme and unit conversion; Figure S6: qPCR, LAMP and LAMP-CRISPR/Cas12a on spiked whole blood in a single representative run; Figure S7: Sequence verification of the dxs target; Figure S8: LAMP and CRISPR/Cas12a read-outs for the same templates side by side; Data S1: Reaction-level data, including a sheet with the Cq and end-point ΔR of all 62 reactions of the limit-of-detection experiment, the specificity panel and the de-identified clinical dataset.

## Abbreviations

The following abbreviations are used in this manuscript:

Abbreviation	Definition
BSA	bovine serum albumin
BSI	bloodstream infection
CI	confidence interval
CFU	colony-forming unit
crRNA	CRISPR RNA
Cq	quantification cycle
IQR	interquartile range
LAMP	loop-mediated isothermal amplification
LOD	limit of detection
NPA	negative percent agreement
NTC	no-template control
PPA	positive percent agreement
qPCR	real-time polymerase chain reaction
RFU	relative fluorescence units
SOFA	Sequential Organ Failure Assessment
TTP	time-to-positivity
