# Supplementary material for: A LAMP–CRISPR/Cas12a Assay for Specific Detection of Escherichia coli in Whole Blood: Analytical Performance and Clinical Evaluation in Suspected Bloodstream Infection
Source: Diagnostics (Basel). 2026 Nov 9;16(21):3692. doi: 10.3390/diagnostics16213692 (PMC13763177; doi:10.3390/diagnostics16213692)
Supplement: Supplementary file 1 [file diagnostics-16-03692-s001.zip › Supplementary_Materials_diagnostics-4669354.pdf]

Supplementary Materials

A LAMP-CRISPR/Cas12a Assay for Specific Detection of *Escherichia coli* in Whole Blood: Analytical Performance and Clinical Evaluation in Suspected Bloodstream Infection

Dao Thanh Quyen, Nguyen Sy Thau, Pham Quang Trung, Nguyen Quang Huy, Le Mai Parkes, Dao Thi Huyen, Phan Quoc Hoan, Do Thu Trang, Le Huu Song and Ngo Tat Trung *

* Correspondence: tatrungngo@gmail.com

Contents

Figure S1. End-point Cas12a fluorescence of the specificity panel.
Figure S2. qPCR standard curves (six independent runs and master curve).
Figure S3. LAMP-CRISPR/Cas12a raw kinetic data and call criterion in the LOD experiments.
Figure S4. *E. coli* qPCR Cq versus procalcitonin and platelet count.
Figure S5. Spiking scheme and unit conversion.
Figure S6. qPCR, LAMP and LAMP-CRISPR/Cas12a on spiked whole blood in a single representative run.
Figure S7. Sequence verification of the dxs target.
Figure S8. LAMP and CRISPR/Cas12a read-outs for the same templates.
Table S1. Specificity panel: results for each isolate and control.
Table S2. Sequence verification of the *dxs* target (BLASTn).
Table S3. qPCR standard-curve parameters by run.
Table S4. Detection rates of qPCR and LAMP-CRISPR/Cas12a in spiked whole blood.
Table S5. Probit analysis (SPSS PROBIT): parameters and estimated concentrations with 95% fiducial limits.
Table S6. Conversion of CFU to *dxs* copies by growth phase and empirical templates per CFU.
Table S7. Reaction-level LAMP-CRISPR/Cas12a results (LOD and patient experiments).
Table S8. Time-to-positivity (TTP) and T50 by experiment and sample group.
Table S9. De-identified per-patient results (n = 52).
Table S10. Agreement between methods: 2 × 2 tables, Cohen's κ and McNemar test.
Table S11. Characteristics of *E. coli* qPCR-positive patients by LAMP-CRISPR/Cas12a and blood-culture result.
Data S1. Data_S1.xlsx — reaction-level LAMP-CRISPR/Cas12a data, specificity panel and de-identified clinical dataset.

Supplementary Figures

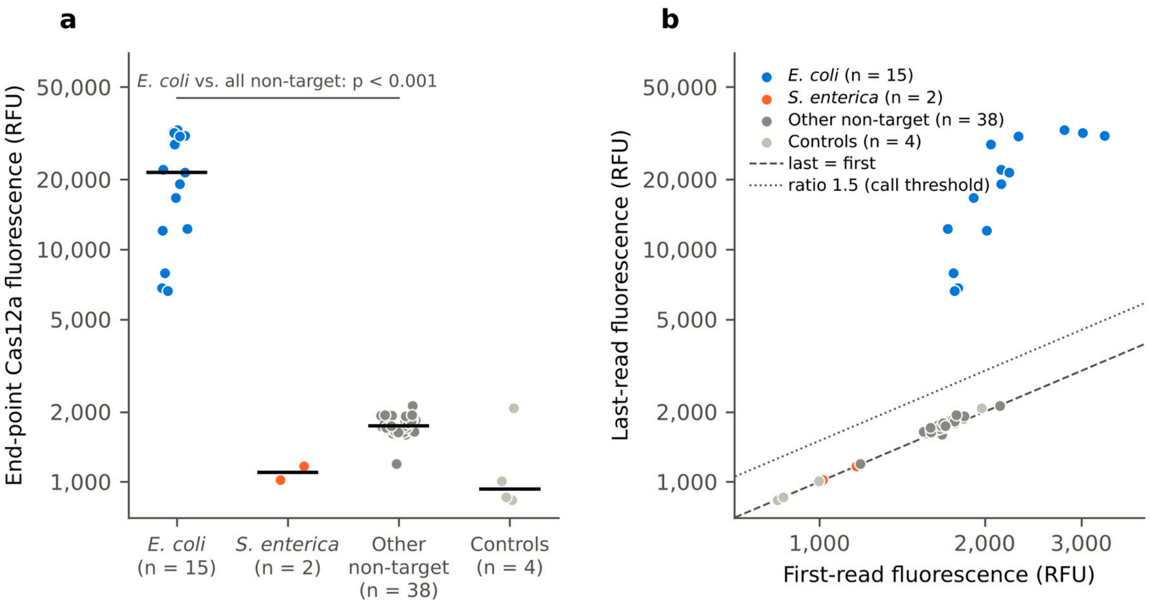

Figure S1. End-point Cas12a fluorescence of the specificity panel (one reaction per sample; raw values in Table S1). (**a**) Last-read fluorescence (RFU, log scale) of 15 *E. coli*, 2 *S. enterica* and 38 other non-target isolates and 4 controls (3 NTC, 1 human DNA); bars, medians. *E. coli*, median 21,501 RFU (IQR 12,075–30,910); non-target isolates, 1734 RFU (1663–1829); Mann-Whitney *U* test (asymptotic), $p < 0.001$. (**b**) First-read versus last-read fluorescence for the same reactions; dashed line, last = first; dotted line, last/first ratio 1.5 (positive call). All 15 *E. coli* lay above the call line (ratio 3.78–13.85), whereas every non-target isolate and control lay on the identity line (0.95–1.09). The two *S. enterica* strains (1170 and 1017 RFU, ratio 1.00) showed no rise above their initial reading.

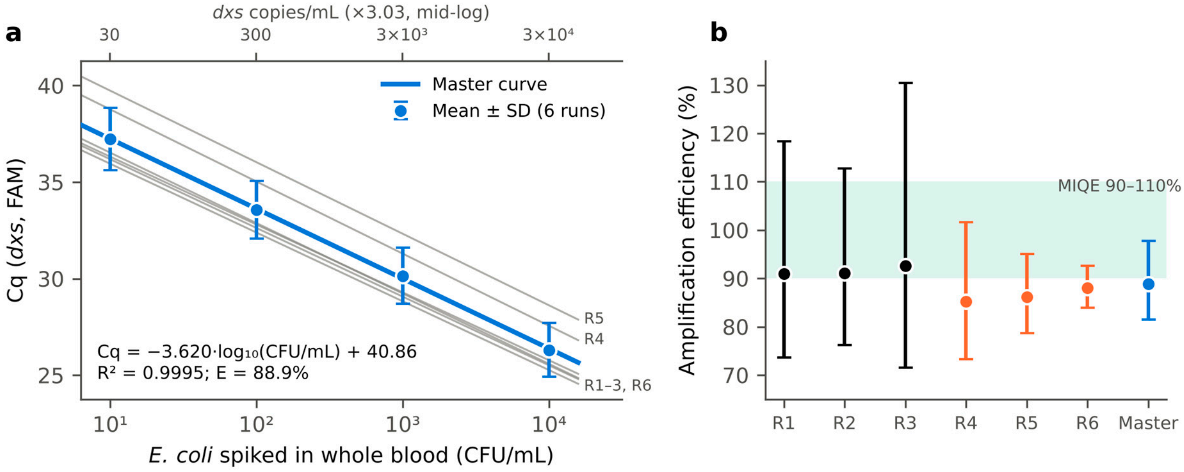

Figure S2. qPCR standard curves for *E. coli dxs* in spiked whole blood. (**a**) Cq versus $\log_{10}$(CFU/mL) for six independent ten-fold dilution series (10^1–10^4 CFU/mL; grey lines, runs R1–R6) and the master curve fitted to the mean Cq (blue; points, mean ± SD of the six runs): Cq = $-3.620 \cdot \log_{10}$(CFU/mL) + 40.86; $R^2 = 0.9995$; efficiency 88.9%. Top axis, *dxs* copies/mL with the growth-phase factor (3.03 copies/CFU). (**b**) Amplification efficiency of each run and of the master curve with 95% CI; shaded band, MIQE acceptance range 90–110%. R1–R3 were within the range (black); R4–R6 were below 90% (orange); master curve 88.9% (95% CI 81.6–97.6). Parameters of each run are given in Table S3.

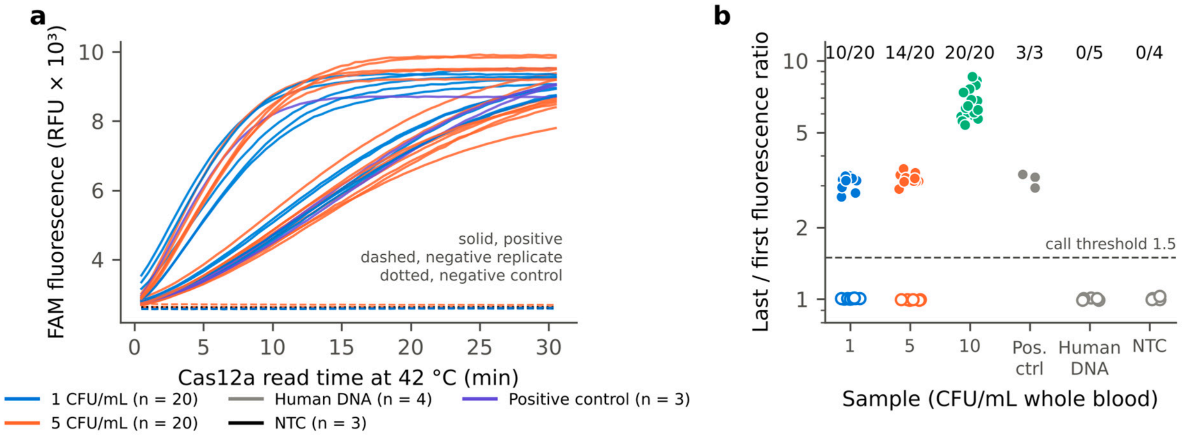

Figure S3. LAMP-CRISPR/Cas12a raw kinetic data in the LOD experiments. (**a**) Cas12a *trans*-cleavage fluorescence of all reactions in LOD run 1 (20 replicates each at 1 and 5 CFU/mL, 4 human-DNA negatives, 3 NTC and 3 positive controls); solid, positive; dashed, negative replicate; dotted, negative control. (**b**) Last/first fluorescence ratio of every reaction in LOD runs 1 and 2 (10 CFU/mL replicates, one further human-DNA negative and NTC from run 2); filled, positive; open, negative; dashed line, call threshold 1.5; numbers, positive/tested. Positive and negative reactions were completely separated (ratios 2.69–8.59 vs. 0.99–1.03). Reaction-level values are listed in Table S7.

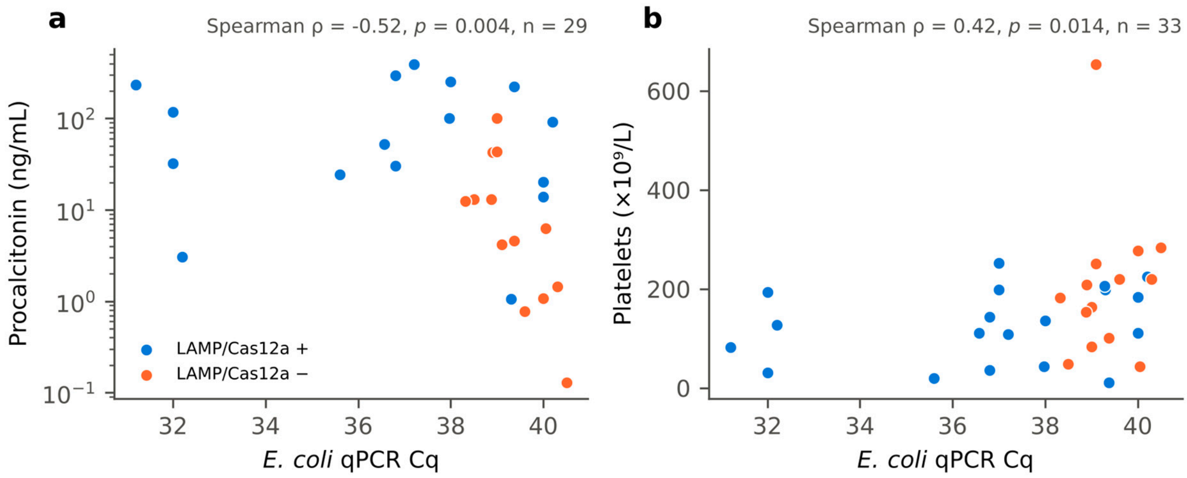

Figure S4. *E. coli* qPCR Cq versus host-response markers in the 33 *E. coli* qPCR-positive patients. (**a**) Procalcitonin (log scale; n = 29), Spearman ϱ = −0.52, p = 0.004. (**b**) Platelet count (n = 33), ϱ = 0.42, p = 0.014. Blue, LAMP-CRISPR/Cas12a positive; orange, LAMP-CRISPR/Cas12a negative. Patient-level values are listed in Table S9.

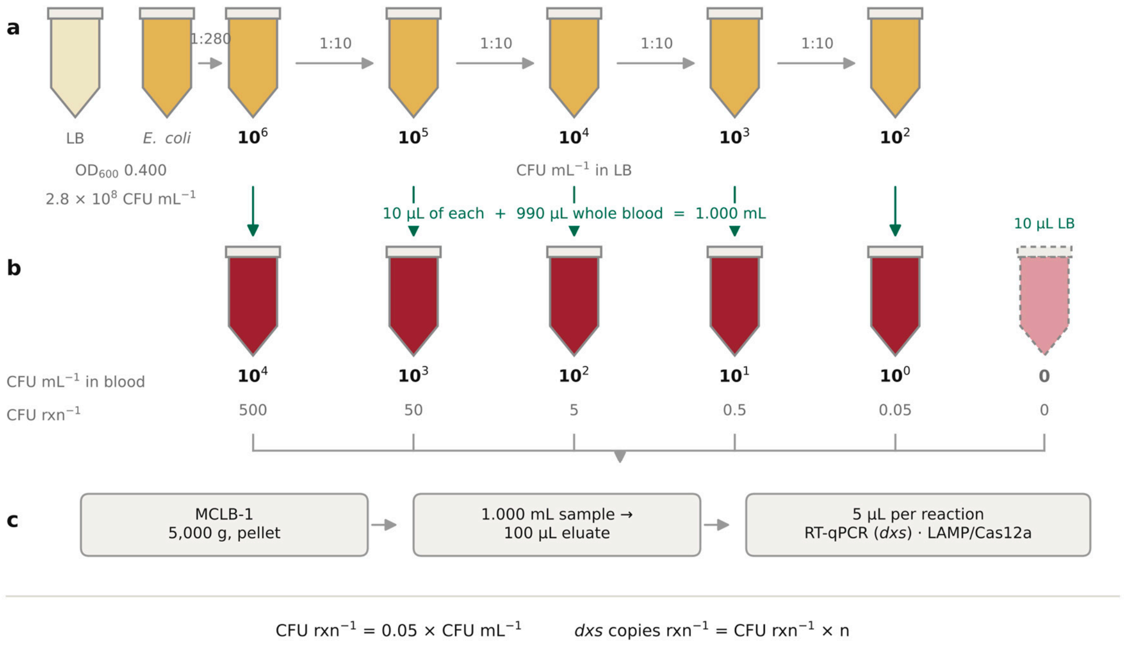

Figure S5. Spiking scheme and unit conversion. (a) Mid-exponential *Escherichia coli* (OD_{600} 0.400; 2.8×10^8 CFU/mL) diluted 1:280 and then ten-fold in LB. (b) 10 µL of each dilution in 990 µL of whole blood gives 10^4–10^0 CFU/mL (500–0.05 CFU per reaction); 10 µL of LB in blood is the human DNA control. (c) Leukocyte depletion, extraction into 100 µL and 5 µL per reaction.

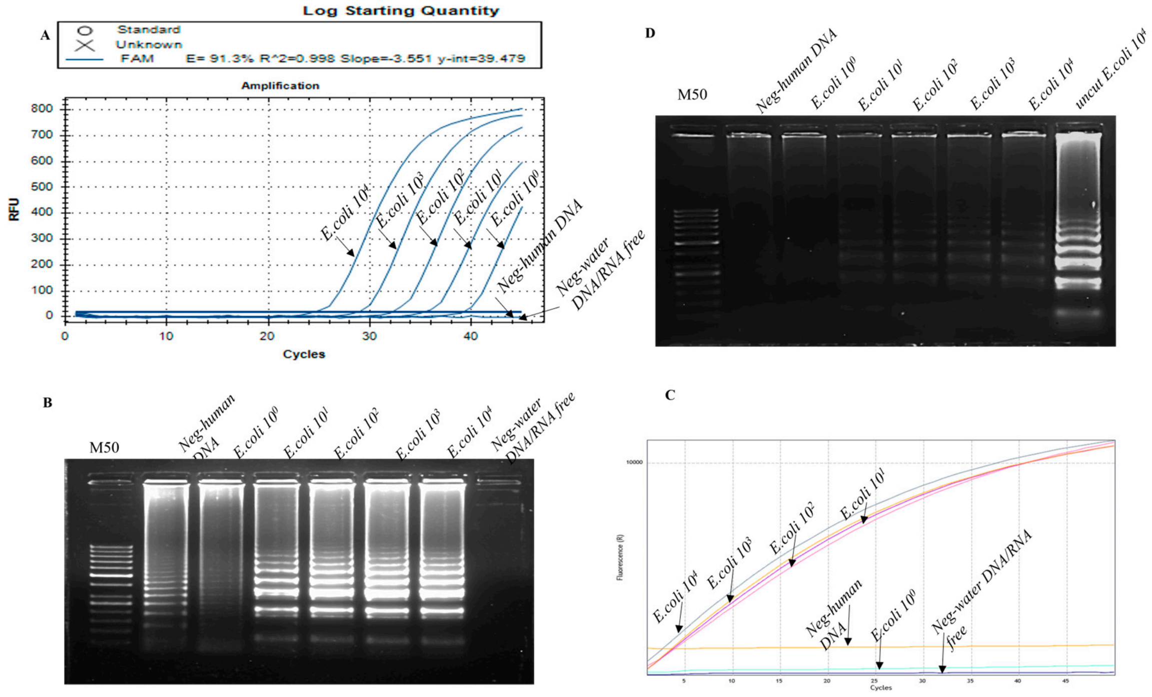

Figure S6. qPCR, LAMP and LAMP-CRISPR/Cas12a on *Escherichia coli*-spiked whole blood (10^4–10^0 CFU/mL) and controls, in a single representative run. (A) qPCR amplification curves; (B) stand-alone LAMP products on a 2% agarose gel; (C) Cas12a fluorescence; (D) Cas12a reaction products on a 2% agarose gel. This is the record of one run in which one set of extracts was carried through every read-out in parallel; no quantitative inference is drawn from it.

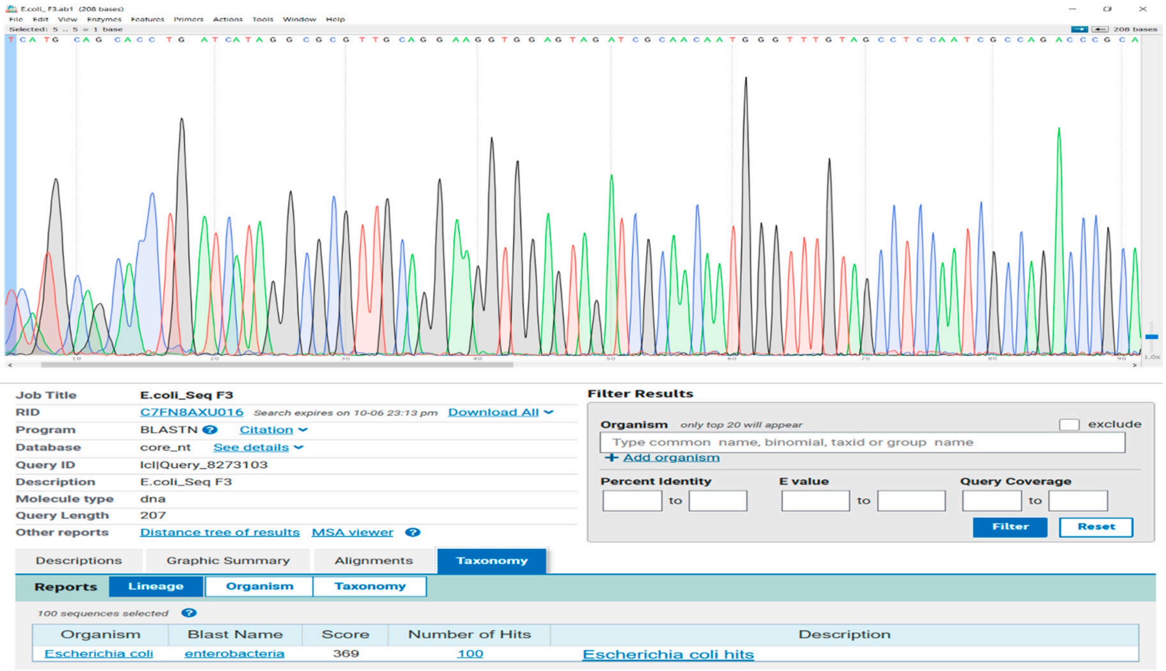

Figure S7. Sequence verification of the dxs target. Sanger sequencing chromatogram of the F3/B3 amplicon of Escherichia coli (read with primer F3; 208 bases) and BLASTn result against the NCBI core_nt database (query length 207 nt): 100% identity, 99% query coverage, bit score 369, E-value 1 × 10^{-97}; all top 100 hits were *Escherichia coli*.

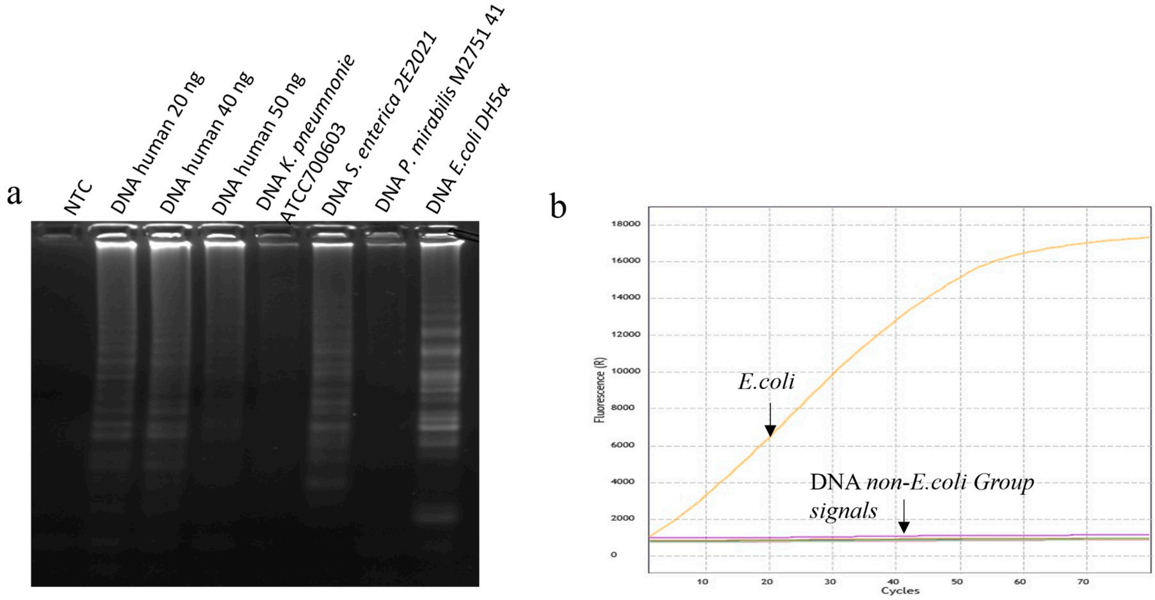

Figure S8. LAMP and CRISPR/Cas12a read-outs for the same templates. Left, agarose gel of LAMP products from the no-template control, human genomic DNA at 20, 40 and 50 ng, three non-target isolates and the Escherichia coli positive control; right, the corresponding Cas12a fluorescence traces. Human genomic DNA produces a ladder that is not distinguishable by morphology from that of the positive control, whereas the Cas12a read-out separates them.

Supplementary Tables

Table S1. Specificity panel: LAMP-CRISPR/Cas12a result for each isolate and control (one reaction per sample).

Panel	Species	Strain	RFU first read	RFU last read	Last/first ratio	Cas12a call	TTP, min	LAMP gel [a]
Target	*Escherichia coli*	ATCC25922	2304	30763	13.35	Positive	0.79	n.t.

Panel	Species	Strain	RFU first read	RFU last read	Last/first ratio	Cas12a call	TTP, min	LAMP gel [a]
Target	*Escherichia coli*	DH5α (K-12)	1907	16678	8.75	Positive	1.79	+
Target	*Escherichia coli*	M2670 35	2216	21501	9.70	Positive	1.26	n.t.
Target	*Escherichia coli*	M4492 252	2140	22101	10.33	Positive	1.32	+
Target	*Escherichia coli*	M4546 257	3016	31756	10.53	Positive	0.73	n.t.
Target	*Escherichia coli*	M4659 264	2050	28406	13.85	Positive	0.97	n.t.
Target	*Escherichia coli*	M4669 265	2794	32767	11.73	Positive	0.73	n.t.
Target	*Escherichia coli*	M4742 270	1752	7919	4.52	Positive	3.60	n.t.
Target	*Escherichia coli*	M6343 99	1712	12249	7.16	Positive	2.69	n.t.
Target	*Escherichia coli*	M7001 103	3305	30910	9.35	Positive	0.71	n.t.
Target	*Escherichia coli*	M8249 107	1760	6647	3.78	Positive	3.72	n.t.
Target	*Escherichia coli*	M8458 110	4684	32049	6.84	Positive	0.65	n.t.
Target	*Escherichia coli*	M8466 111	1788	6812	3.81	Positive	4.21	n.t.
Target	*Escherichia coli*	M8473 109	2139	19149	8.95	Positive	1.52	+
Target	*Escherichia coli*	M897 17	2014	12075	5.99	Positive	2.19	n.t.
Non-target	*Acinetobacter baumannii*	M4577 258	1832	1873	1.02	Negative	–	n.t.
Non-target	*Acinetobacter baumannii*	M4675 91	1683	1713	1.02	Negative	–	±
Non-target	*Acinetobacter baumannii*	M4908 279	1642	1679	1.02	Negative	–	n.t.
Non-target	*Acinetobacter baumannii*	M690 11	1605	1645	1.02	Negative	–	n.t.
Non-target	*Candida albicans*	ATCC 14053	1723	1777	1.03	Negative	–	n.t.
Non-target	*Candida albicans*	M4126 230	1643	1749	1.06	Negative	–	n.t.
Non-target	*Enterobacter cloacae*	M3205 66	1646	1696	1.03	Negative	–	n.t.
Non-target	*Enterococcus faecalis*	M2945 56	1729	1844	1.07	Negative	–	n.t.
Non-target	*Enterococcus faecium*	M6653 381	1714	1772	1.03	Negative	–	n.t.
Non-target	*Klebsiella aerogenes*	M108 5	1758	1826	1.04	Negative	–	n.t.
Non-target	*Klebsiella oxytoca*	M3354 190	1598	1631	1.02	Negative	–	–
Non-target	*Klebsiella pneumoniae*	ATCC 700603	1678	1791	1.07	Negative	–	–
Non-target	*Klebsiella pneumoniae*	M3013 61	1655	1686	1.02	Negative	–	n.t.
Non-target	*Klebsiella pneumoniae*	M3062 64	1658	1709	1.03	Negative	–	n.t.
Non-target	*Klebsiella pneumoniae*	M3154 69	1680	1720	1.02	Negative	–	n.t.
Non-target	*Klebsiella pneumoniae*	M3687 50	1834	1922	1.05	Negative	–	–
Non-target	*Klebsiella pneumoniae*	M3900 10	2131	2118	0.99	Negative	–	n.t.
Non-target	*Klebsiella pneumoniae*	M41107 54	1546	1640	1.06	Negative	–	n.t.
Non-target	*Klebsiella pneumoniae*	M4434 21	1670	1594	0.95	Negative	–	n.t.
Non-target	*Klebsiella pneumoniae*	M59 2	1764	1786	1.01	Negative	–	–
Non-target	*Listeria monocytogenes*	124	1592	1705	1.07	Negative	–	n.t.
Non-target	*Neisseria meningitidis*	serogroup B	1761	1824	1.04	Negative	–	n.t.
Non-target	*Proteus mirabilis*	M2751 41	1796	1933	1.08	Negative	–	–
Non-target	*Pseudomonas aeruginosa*	ATCC 27853	1771	1830	1.03	Negative	–	n.t.
Non-target	*Pseudomonas aeruginosa*	M3043 62	1559	1616	1.04	Negative	–	n.t.
Non-target	*Pseudomonas aeruginosa*	M6853 389	1616	1657	1.03	Negative	–	n.t.
Non-target	*Salmonella enterica*	2E2021	1013	1017	1.00	Negative	–	±
Non-target	*Salmonella enterica*	M8261 105	1168	1170	1.00	Negative	–	n.t.
Non-target	*Staphylococcus aureus*	ATCC 29213	1694	1738	1.03	Negative	–	n.t.
Non-target	*Staphylococcus aureus*	M2743 42	1783	1928	1.08	Negative	–	n.t.
Non-target	*Staphylococcus aureus*	M2856 48	1773	1941	1.09	Negative	–	n.t.
Non-target	*Staphylococcus aureus*	M3283 72	1749	1857	1.06	Negative	–	n.t.
Non-target	*Staphylococcus aureus*	M3286 71	1666	1743	1.05	Negative	–	n.t.
Non-target	*Staphylococcus aureus*	M3861 78	1645	1723	1.05	Negative	–	n.t.
Non-target	*Staphylococcus aureus*	M5085 95	1610	1686	1.05	Negative	–	n.t.
Non-target	*Staphylococcus aureus*	M5098 94	1187	1192	1.00	Negative	–	n.t.
Non-target	*Staphylococcus aureus*	M8358 106	1760	1927	1.09	Negative	–	n.t.
Non-target	*Staphylococcus hominis*	M2822 49	1608	1634	1.02	Negative	–	n.t.
Non-target	*Streptococcus agalactiae*	M2967 55	1621	1730	1.07	Negative	–	n.t.
Non-target	*Streptococcus suis*	M6311 98	1756	1828	1.04	Negative	–	n.t.

Panel	Species	Strain	RFU first read	RFU last read	Last/first ratio	Cas12a call	TTP, min	LAMP gel [a]
Control	Human genomic DNA	–	1974	2070	1.05	Negative	–	–
Control	No-template control	–	997	1006	1.01	Negative	–	n.t.
Control	No-template control	–	839	836	1.00	Negative	–	n.t.
Control	No-template control	–	858	856	1.00	Negative	–	n.t.

Positive, last/first fluorescence ratio ≥ 1.5. TTP, time at which baseline-corrected fluorescence exceeded 10 × SD of the negative-control signal (– , not reached within 25–30 min). [a] Stand-alone LAMP on 2% agarose gel for the 11 samples shown in Figure 5c: +, ladder (more than three discrete bands with high-molecular-weight material at or near the well); ±, partial ladder or smear (three or fewer discrete bands, or continuous fluorescence without discrete bands); −, no product; n.t., not tested on gel. Inclusivity 15/15 (100%; Wilson 95% CI 79.6–100); exclusivity 40/40 (100%; 91.2–100).

Table S2. Sequence verification of the *dxs* target by Sanger sequencing and BLASTn (NCBI core_nt).

Item	Result
Template / primers	*E. coli* DNA; outer LAMP primers F3/B3 (amplicon 243 bp, NC_000913.3 438,921–439,163)
Sequencing	Sanger, primer F3, Applied Biosystems 3500 Genetic Analyzer; trace length 208 bases
BLASTn query length	207 nt
Best match	*Escherichia coli dxs* (1-deoxy-D-xylulose-5-phosphate synthase)
Identity / query coverage	100% / 99%
Bit score / E-value	369 / 1×10^{-97}
Taxonomy of top 100 hits	100/100 *Escherichia coli* (Enterobacteriaceae)
crRNA spacer vs. *Salmonella enterica*	No significant similarity (BLASTn)

Chromatogram and BLASTn summary are shown in Figure 6 of the main text.

Table S3. qPCR standard-curve parameters by run (Cq = slope·$\log_{10}$(CFU/mL) + intercept).

Run	Slope	Intercept	R^2	Efficiency, %
Run 1	-3.561	39.51	0.9968	90.9
Run 2	-3.557	39.74	0.9978	91.0
Run 3	-3.514	39.83	0.9950	92.6
Run 4	-3.735	42.50	0.9984	85.2
Run 5	-3.705	43.44	0.9995	86.2
Run 6	-3.646	40.17	0.9999	88.0
Mean ± SD	-3.620 ± 0.089	40.87 ± 1.67	–	89.0 ± 3.0
Master curve (mean Cq)	−3.620	40.86	0.9995	88.9 (95% CI 81.6–97.6)

Efficiency = 10^(−1/slope) − 1. Mean (SD) Cq of the six runs: 10^1 CFU/mL, 37.24 (1.61); 10^2 CFU/mL, 33.56 (1.50); 10^3 CFU/mL, 30.15 (1.46); 10^4 CFU/mL, 26.31 (1.39).

Table S4. Detection rates of qPCR and LAMP-CRISPR/Cas12a in *E. coli*-spiked whole blood.

Spike, CFU/mL	CFU/reaction	qPCR, n/N (%; Wilson 95% CI)	LAMP–CRISPR/Cas12a, n/N (%; Wilson 95% CI)
10 000	500	2/2 (100; 34.2–100.0)	2/2 (100; 34.2–100.0)
1 000	50	2/2 (100; 34.2–100.0)	2/2 (100; 34.2–100.0)
100	5	6/6 (100; 61.0–100.0)	6/6 (100; 61.0–100.0)
10	0.5	20/20 (100; 83.9–100.0)	20/20 (100; 83.9–100.0)
5	0.25	18/20 (90; 69.9–97.2)	14/20 (70; 48.1–85.5)
1	0.05	15/20 (75; 53.1–88.8)	10/20 (50; 29.9–70.1)
Negative controls [a]	0	0/4	0/9

Independently extracted replicates; 5 µL of 100 µL eluate per reaction (1 CFU/mL = 0.05 CFU/reaction). Observed LOD (≥ 95% detection, ≥ 19/20) = 10 CFU/mL for both assays. [a] Human-DNA negatives, NTC and matrix blank.

Table S5. Probit analysis of detection in spiked whole blood (IBM SPSS Statistics 20, PROBIT procedure, $\log_{10}$ concentration, all six levels).

Parameter	qPCR	LAMP–CRISPR/Cas12a

Parameter	qPCR	LAMP–CRISPR/Cas12a
Intercept a (SE)	0.626 (0.298)	-0.113 (0.274)
Slope b per $\log_{10}$ CFU/mL (SE)	1.339 (0.567)	1.526 (0.450)
Slope Z test, *p*	0.018	0.001
Pearson χ^2 (df); *p*	1.134 (4); 0.889	4.274 (4); 0.370
Heterogeneity factor applied	No	No
LOD1, CFU/mL (95% fiducial limits)	< 0.01 (< 0.01–0.09)	0.04 (< 0.01–0.19)
LOD5, CFU/mL (95% fiducial limits)	0.02 (< 0.01–0.18)	0.10 (< 0.01–0.36)
LOD10, CFU/mL (95% fiducial limits)	0.04 (< 0.01–0.26)	0.17 (< 0.01–0.52)
LOD20, CFU/mL (95% fiducial limits)	0.08 (< 0.01–0.40)	0.33 (0.02–0.82)
LOD30, CFU/mL (95% fiducial limits)	0.14 (< 0.01–0.55)	0.54 (0.05–1.14)
LOD40, CFU/mL (95% fiducial limits)	0.22 (< 0.01–0.73)	0.81 (0.12–1.55)
LOD50, CFU/mL (95% fiducial limits)	0.34 (< 0.01–0.95)	1.19 (0.29–2.12)
LOD60, CFU/mL (95% fiducial limits)	0.53 (< 0.01–1.28)	1.74 (0.62–3.05)
LOD70, CFU/mL (95% fiducial limits)	0.84 (< 0.01–1.84)	2.62 (1.28–5.07)
LOD80, CFU/mL (95% fiducial limits)	1.45 (0.10–3.45)	4.22 (2.40–11.4)
LOD90, CFU/mL (95% fiducial limits)	3.09 (1.26–36.3)	8.20 (4.40–45.6)
LOD_{95}, CFU/mL (95% fiducial limits)	5.77 (2.61–957.7)	14.19 (6.66–156.3)
LOD99, CFU/mL (95% fiducial limits)	18.6 (5.94–763 077)	39.7 (13.5–1 685)
Empirical templates per CFU, k (Wald 95% CI) [a]	15.4 (10.0–23.7)	7.4 (5.1–10.8)

Model P(detection) = $\Phi(a + b\cdot\log_{10}C)$. 95% fiducial limits by Fieller's method; a heterogeneity factor is applied by SPSS when the Pearson χ^2 significance is < 0.15. As a check, the authors' original SPSS 20 run restricted to the three 20-replicate levels (1, 5 and 10 CFU/mL) gave LOD_{95} = 14.38 CFU/mL (95% fiducial limits 6.64–192.7; Pearson χ^2 p = 0.04), consistent with the six-level estimate. [a] Generalized linear model, binomial distribution, complementary log–log link, offset ln(0.05 × C).

Table S6. Conversion of the probit LOD_{95} from CFU to *dxs* copies by growth phase of the spike culture (Cooper–Helmstetter model; *dxs* at relative position m = 0.50 between *oriC* and *ter*; C = 40 min, D = 20 min).

Growth phase	Condition	τ, min	*dxs* copies/CFU	qPCR LOD_{95}, copies/mL (per reaction)	LAMP-CRISPR/Cas12a LOD_{95}, copies/mL (per reaction)
Fast exponential	LB 37 °C, OD_{600} 0.2–0.5	20	4.00	23.1 (1.15)	56.7 (2.83)
Mid-exponential (spike culture, OD_{600} 0.400)	LB 37 °C	25	3.03	17.5 (0.87)	43.0 (2.15)
Late exponential	OD_{600} ≈ 1	40	2.00	11.5 (0.58)	28.4 (1.42)
Slow growth	minimal medium	60	1.59	9.2 (0.46)	22.5 (1.13)
Stationary	≥ 16 h overnight	–	1–2	5.8–11.5 (0.29–0.58)	14.2–28.4 (0.71–1.42)
Empirical Poisson (data-derived) [a]	single-hit model fitted to qPCR LOD data	–	15.4 (10.0–23.7)	89 (4.4)	218 (10.9)

$n = 2^{[(C(1-m)+D)/\tau]}$; *dxs*, NC_000913.3 438,315–440,177; *oriC* 3,925,744. LOD_{95} = 5.77 (qPCR) and 14.19 CFU/mL (LAMP–CRISPR/Cas12a) from Table S5; per reaction = copies/mL × 0.05 mL. Values identical to main Table 5. [a] Amplifiable templates per nominal CFU, *k* (95% CI), estimated from the qPCR detection rates (Table S5); applied to both assays. The corresponding estimate from the LAMP-CRISPR/Cas12a detection rates was 7.4 (5.1–10.8).

Table S7. Reaction-level LAMP-CRISPR/Cas12a results in the LOD and patient experiments (*n* = 143 reactions; sample names as recorded, patients by study number).

Experiment	Sample name	Group	RFU first read	RFU last read	Last/first ratio	Call	TTP, min	T50, min
LOD run 1 (1 and 5 CFU/mL)	*E.coli*5	5 CFU/mL	2684	7796	2.90	Positive	1.84	13.2
LOD run 1 (1 and 5 CFU/mL)	*E.coli*1	1 CFU/mL	3538	9508	2.69	Positive	0.73	4.1
LOD run 1 (1 and 5 CFU/mL)	*E.coli*1	1 CFU/mL	2598	2608	1.00	Negative	–	–
LOD run 1 (1 and 5 CFU/mL)	*E.coli*1	1 CFU/mL	2883	9061	3.14	Positive	0.95	6.4
LOD run 1 (1 and 5 CFU/mL)	*E.coli*5	5 CFU/mL	2612	2608	1.00	Negative	–	–
LOD run 1 (1 and 5 CFU/mL)	*E.coli*5	5 CFU/mL	3017	9527	3.16	Positive	0.74	4.5
LOD run 1 (1 and 5 CFU/mL)	*E.coli*5	5 CFU/mL	2760	8588	3.11	Positive	1.66	13.3

Experiment	Sample name	Group	RFU first read	RFU last read	Last/first ratio	Call	TTP, min	T50, min
LOD run 1 (1 and 5 CFU/mL)	*E.coli*1	1 CFU/mL	2583	2604	1.01	Negative	–	–
LOD run 1 (1 and 5 CFU/mL)	*E.coli*1	1 CFU/mL	2580	2602	1.01	Negative	–	–
LOD run 1 (1 and 5 CFU/mL)	*E.coli*1	1 CFU/mL	2817	8930	3.17	Positive	1.52	12.0
LOD run 1 (1 and 5 CFU/mL)	*E.coli*5	5 CFU/mL	2763	8631	3.12	Positive	1.83	14.0
LOD run 1 (1 and 5 CFU/mL)	*E.coli*5	5 CFU/mL	2739	8680	3.17	Positive	1.73	13.5
LOD run 1 (1 and 5 CFU/mL)	*E.coli*5	5 CFU/mL	2713	2684	0.99	Negative	–	–
LOD run 1 (1 and 5 CFU/mL)	*E.coli*1	1 CFU/mL	2603	2612	1.00	Negative	–	–
LOD run 1 (1 and 5 CFU/mL)	*E.coli*1	1 CFU/mL	2820	8953	3.17	Positive	1.61	12.4
LOD run 1 (1 and 5 CFU/mL)	*E.coli*1	1 CFU/mL	2574	2592	1.01	Negative	–	–
LOD run 1 (1 and 5 CFU/mL)	*E.coli*5	5 CFU/mL	2739	8546	3.12	Positive	2.21	15.1
LOD run 1 (1 and 5 CFU/mL)	*E.coli*5	5 CFU/mL	2766	9189	3.32	Positive	1.71	13.8
LOD run 1 (1 and 5 CFU/mL)	*E.coli*5	5 CFU/mL	2885	9377	3.25	Positive	1.43	11.9
LOD run 1 (1 and 5 CFU/mL)	*E.coli*1	1 CFU/mL	3151	9276	2.94	Positive	0.85	5.7
LOD run 1 (1 and 5 CFU/mL)	*E.coli*1	1 CFU/mL	2565	2587	1.01	Negative	–	–
LOD run 1 (1 and 5 CFU/mL)	*E.coli*1	1 CFU/mL	2569	2572	1.00	Negative	–	–
LOD run 1 (1 and 5 CFU/mL)	*E.coli*5	5 CFU/mL	2614	2612	1.00	Negative	–	–
LOD run 1 (1 and 5 CFU/mL)	*E.coli*5	5 CFU/mL	2633	2623	1.00	Negative	–	–
LOD run 1 (1 and 5 CFU/mL)	*E.coli*1	1 CFU/mL	2742	9052	3.30	Positive	1.99	14.2
LOD run 1 (1 and 5 CFU/mL)	*E.coli*1	1 CFU/mL	2719	8745	3.22	Positive	1.95	14.1
LOD run 1 (1 and 5 CFU/mL)	Ne-human DNA	Human DNA	2607	2641	1.01	Negative	–	–
LOD run 1 (1 and 5 CFU/mL)	*E.coli*Positive	Positive control	2706	9063	3.35	Positive	1.77	13.9
LOD run 1 (1 and 5 CFU/mL)	*E.coli*5	5 CFU/mL	2684	8396	3.13	Positive	1.91	14.3
LOD run 1 (1 and 5 CFU/mL)	*E.coli*5	5 CFU/mL	2619	2599	0.99	Negative	–	–
LOD run 1 (1 and 5 CFU/mL)	Ne-human	Human DNA	2629	2610	0.99	Negative	–	–
LOD run 1 (1 and 5 CFU/mL)	*E.coli*1	1 CFU/mL	3340	9335	2.79	Positive	0.75	4.4
LOD run 1 (1 and 5 CFU/mL)	*E.coli*1	1 CFU/mL	2572	2595	1.01	Negative	–	–
LOD run 1 (1 and 5 CFU/mL)	Ne-human DNA	Human DNA	2573	2594	1.01	Negative	–	–
LOD run 1 (1 and 5 CFU/mL)	*E.coli*Positive	Positive control	2777	9031	3.25	Positive	2.08	15.0
LOD run 1 (1 and 5 CFU/mL)	*E.coli*5	5 CFU/mL	2796	9836	3.52	Positive	0.81	5.9
LOD run 1 (1 and 5 CFU/mL)	*E.coli*5	5 CFU/mL	2606	2600	1.00	Negative	–	–
LOD run 1 (1 and 5 CFU/mL)	*E.coli*Positive	Positive control	2961	8702	2.94	Positive	0.74	4.3
LOD run 1 (1 and 5 CFU/mL)	H20-NTC	NTC	2617	2606	1.00	Negative	–	–
LOD run 1 (1 and 5 CFU/mL)	*E.coli*1	1 CFU/mL	2821	9255	3.28	Positive	0.90	6.5
LOD run 1 (1 and 5 CFU/mL)	*E.coli*1	1 CFU/mL	2570	2587	1.01	Negative	–	–
LOD run 1 (1 and 5 CFU/mL)	Ne-human DNA	Human DNA	2575	2598	1.01	Negative	–	–
LOD run 1 (1 and 5 CFU/mL)	H20-NTC	NTC	2594	2618	1.01	Negative	–	–
LOD run 1 (1 and 5 CFU/mL)	*E.coli*5	5 CFU/mL	2930	9493	3.24	Positive	0.76	5.0
LOD run 1 (1 and 5 CFU/mL)	*E.coli*5	5 CFU/mL	2906	9894	3.40	Positive	0.82	5.9
LOD run 1 (1 and 5 CFU/mL)	H20-NTC	NTC	2609	2595	0.99	Negative	–	–
LOD run 1 (1 and 5 CFU/mL)	*E.coli*1	1 CFU/mL	2573	2604	1.01	Negative	–	–
LOD run 1 (1 and 5 CFU/mL)	*E.coli*1	1 CFU/mL	2770	8728	3.15	Positive	1.75	13.7
LOD run 1 (1 and 5 CFU/mL)	*E.coli*5	5 CFU/mL	2862	9203	3.22	Positive	0.79	5.3
LOD run 1 (1 and 5 CFU/mL)	*E.coli*5	5 CFU/mL	2729	8480	3.11	Positive	1.60	12.5
LOD run 2 (10 CFU/mL) and patient run B	*E.coli*10	10 CFU/mL	1107	9135	8.25	Positive	0.99	6.4
LOD run 2 (10 CFU/mL) and patient run B	*E.coli*10	10 CFU/mL	1911	11278	5.90	Positive	1.06	8.9
LOD run 2 (10 CFU/mL) and patient run B	Patient 5	Patient	1595	9935	6.23	Positive	1.24	9.4
LOD run 2 (10 CFU/mL) and patient run B	*E.coli*10	10 CFU/mL	1571	9728	6.19	Positive	1.23	9.9
LOD run 2 (10 CFU/mL) and patient run B	*E.coli*10	10 CFU/mL	1862	11310	6.08	Positive	1.01	8.3
LOD run 2 (10 CFU/mL) and patient run B	Patient 3	Patient	1058	1122	1.06	Negative	–	–

Experiment	Sample name	Group	RFU first read	RFU last read	Last/first ratio	Call	TTP, min	T50, min
LOD run 2 (10 CFU/mL) and patient run B	*E.coli*10	10 CFU/mL	1028	6488	6.31	Positive	2.09	13.8
LOD run 2 (10 CFU/mL) and patient run B	*E.coli*10	10 CFU/mL	1373	10847	7.90	Positive	1.36	11.1
LOD run 2 (10 CFU/mL) and patient run B	*E.coli*10	10 CFU/mL	1988	11369	5.72	Positive	1.02	8.5
LOD run 2 (10 CFU/mL) and patient run B	Patient 9	Patient	2589	11801	4.56	Positive	0.63	2.9
LOD run 2 (10 CFU/mL) and patient run B	*E.coli*10	10 CFU/mL	2096	12224	5.83	Positive	0.95	8.4
LOD run 2 (10 CFU/mL) and patient run B	*E.coli*10	10 CFU/mL	1677	11427	6.82	Positive	1.12	9.7
LOD run 2 (10 CFU/mL) and patient run B	*E.coli*10	10 CFU/mL	1867	11322	6.07	Positive	1.06	9.1
LOD run 2 (10 CFU/mL) and patient run B	Patient 10	Patient	1476	11220	7.60	Positive	1.31	10.9
LOD run 2 (10 CFU/mL) and patient run B	*E.coli*10	10 CFU/mL	1392	10584	7.61	Positive	1.41	11.6
LOD run 2 (10 CFU/mL) and patient run B	*E.coli*10	10 CFU/mL	1808	10137	5.61	Positive	1.14	9.4
LOD run 2 (10 CFU/mL) and patient run B	*E.coli*10	10 CFU/mL	1891	11496	6.08	Positive	1.02	8.7
LOD run 2 (10 CFU/mL) and patient run B	Patient 21	Patient	1396	11960	8.57	Positive	0.85	5.8
LOD run 2 (10 CFU/mL) and patient run B	*E.coli*10	10 CFU/mL	1891	12011	6.35	Positive	1.02	9.3
LOD run 2 (10 CFU/mL) and patient run B	*E.coli*10	10 CFU/mL	1761	10955	6.22	Positive	1.10	9.4
LOD run 2 (10 CFU/mL) and patient run B	*E.coli*10	10 CFU/mL	2217	11929	5.38	Positive	0.66	3.7
LOD run 2 (10 CFU/mL) and patient run B	Patient 28	Patient	2745	11875	4.33	Positive	0.62	2.7
LOD run 2 (10 CFU/mL) and patient run B	*E.coli*10	10 CFU/mL	1110	7619	6.86	Positive	2.23	14.2
LOD run 2 (10 CFU/mL) and patient run B	*E.coli*10	10 CFU/mL	1814	11718	6.46	Positive	1.03	9.0
LOD run 2 (10 CFU/mL) and patient run B	H20-NTC	NTC	1016	1048	1.03	Negative	–	–
LOD run 2 (10 CFU/mL) and patient run B	Patient 29	Patient	984	10578	10.75	Positive	0.92	5.9
LOD run 2 (10 CFU/mL) and patient run B	*E.coli*10	10 CFU/mL	1237	10628	8.59	Positive	1.64	12.9
LOD run 2 (10 CFU/mL) and patient run B	*E.coli*10	10 CFU/mL	1429	10559	7.39	Positive	1.30	11.0
LOD run 2 (10 CFU/mL) and patient run B	Human DNA	Human DNA	1080	1067	0.99	Negative	–	–
LOD run 2 (10 CFU/mL) and patient run B	Patient 31	Patient	1209	12328	10.20	Positive	1.04	7.6
Patient run A	Patient 27	Patient	2647	2616	0.99	Negative	–	–
Patient run A	H20-NTC	NTC	2767	2754	1.00	Negative	–	–
Patient run A	Patient 3	Patient	2679	2680	1.00	Negative	–	–
Patient run A	Patient 23	Patient	2611	2583	0.99	Negative	–	–
Patient run A	Patient 6	Patient	2856	8975	3.14	Positive	0.81	7.4
Patient run A	Patient 32	Patient	2681	2654	0.99	Negative	–	–
Patient run A	Ne-Human DNA	Human DNA	2741	2740	1.00	Negative	–	–
Patient run A	Patient 15	Patient	2670	2663	1.00	Negative	–	–
Patient run A	Patient 24	Patient	2645	2630	0.99	Negative	–	–
Patient run A	Non-cohort sample	Patient	2677	2632	0.98	Negative	–	–
Patient run A	Patient 38	Patient	2751	2676	0.97	Negative	–	–

Experiment	Sample name	Group	RFU first read	RFU last read	Last/first ratio	Call	TTP, min	T50, min
Patient run A	Patient 33	Patient	2726	2719	1.00	Negative	–	–
Patient run A	Patient 16	Patient	2718	2703	0.99	Negative	–	–
Patient run A	Patient 25	Patient	2639	2604	0.99	Negative	–	–
Patient run A	Patient 7	Patient	2719	8450	3.11	Positive	2.34	14.6
Patient run A	*E.coli*1	1 CFU/mL	2774	2716	0.98	Negative	–	–
Patient run A	Patient 34	Patient	2716	2711	1.00	Negative	–	–
Patient run A	Patient 2	Patient	2754	11791	4.28	Positive	1.18	10.8
Patient run A	Patient 26	Patient	2649	2617	0.99	Negative	–	–
Patient run A	Patient 8	Patient	2673	2631	0.98	Negative	–	–
Patient run A	*E.coli*10	10 CFU/mL	2770	4475	1.62	Positive	4.34	16.3
Patient run A	Patient 35	Patient	2721	2712	1.00	Negative	–	–
Patient run A	Patient 17	Patient	2733	2727	1.00	Negative	–	–
Patient run A	Patient 30	Patient	2651	2614	0.99	Negative	–	–
Patient run A	Patient 11	Patient	2687	2646	0.98	Negative	–	–
Patient run A	*E.coli*100	100 CFU/mL	2781	5392	1.94	Positive	2.74	15.6
Patient run A	Patient 36	Patient	2696	2683	0.99	Negative	–	–
Patient run A	Patient 18	Patient	2670	2656	0.99	Negative	–	–
Patient run A	Patient 1	Patient	3121	10001	3.20	Positive	0.74	6.3
Patient run A	Patient 13	Patient	2654	2627	0.99	Negative	–	–
Patient run A	H20-NTC	NTC	2548	2513	0.99	Negative	–	–
Patient run A	Patient 37	Patient	2786	2782	1.00	Negative	–	–
Patient run A	Patient 19	Patient	2681	2675	1.00	Negative	–	–
Patient run A	Non-cohort sample	Patient	2614	2579	0.99	Negative	–	–
Patient run A	Patient 14	Patient	2651	2625	0.99	Negative	–	–
Patient run A	Patient 12	Patient	2703	2690	1.00	Negative	–	–
Patient run A	Patient 22	Patient	2715	2708	1.00	Negative	–	–
Patient run A	Patient 4	Patient	2616	2576	0.98	Negative	–	–
Patient run A	Patient 20	Patient	2633	2597	0.99	Negative	–	–
Patient run C	Patient 14	Patient	1704	10520	6.18	Positive	0.59	5.0
Patient run C	Patient 47	Patient	1098	5530	5.04	Positive	2.52	14.9
Patient run C	Patient 40	Patient	1066	1076	1.01	Negative	–	–
Patient run C	Patient 42	Patient	1155	10288	8.91	Positive	0.65	6.8
Patient run C	Patient 46	Patient	946	955	1.01	Negative	–	–
Patient run C	Patient 39	Patient	989	994	1.00	Negative	–	–
Patient run C	Patient 49	Patient	979	1003	1.02	Negative	–	–
Patient run C	Non-cohort sample	Patient	948	966	1.02	Negative	–	–
Patient run C	H20	NTC	904	905	1.00	Negative	–	–
Patient run C	Non-cohort sample	Patient	1008	998	0.99	Negative	–	–
Patient run C	Patient 45	Patient	889	977	1.10	Negative	–	–
Patient run C	HUMAN	Human DNA	900	915	1.02	Negative	–	–
Patient run C	Patient 52	Patient	990	978	0.99	Negative	–	–
Patient run C	Patient 44	Patient	1692	12031	7.11	Positive	0.63	6.4
Patient run C	E0	1 CFU/mL	1003	1039	1.04	Negative	–	–
Patient run C	Patient 51	Patient	1011	1009	1.00	Negative	–	–
Patient run C	Patient 43	Patient	1050	9733	9.27	Positive	1.20	11.9
Patient run C	*E.coli*10 pos	10 CFU/mL	1500	7871	5.25	Positive	0.96	10.7
Patient run C	Patient 50	Patient	943	948	1.01	Negative	–	–
Patient run C	Non-cohort sample	Patient	949	1039	1.09	Negative	–	–
Patient run C	Patient 45	Patient	1456	10632	7.30	Positive	0.68	7.0
Patient run C	Patient 48	Patient	944	929	0.98	Negative	–	–
Patient run C	Patient 41	Patient	932	9159	9.83	Positive	0.99	10.1
Patient run C	Patient 46	Patient	1319	6069	4.60	Positive	1.17	11.3

Positive, last/first fluorescence ratio ≥ 1.5. TTP threshold per experiment: 10 × SD of negative-control ΔR (minimum 100 RFU; 100–368 RFU across runs). T50, time to half of the maximal signal above the negative-control baseline. –, not reached. Patients 3, 14, 45 and 46

were tested twice (see Table S9); Non-cohort sample, a patient sample tested in the same run but not part of the 52-patient cohort (shown for completeness, excluded from all analyses). Spiked samples (1–100 CFU/mL) and the matrix blank in the patient runs were run controls.

Table S8. TTP and T50 of LAMP-CRISPR/Cas12a by experiment and sample group.

Experiment	Group	Positive/tested	TTP, min, median (IQR)	T50, min, median (IQR)	T50 range, min	Median ratio	TTP threshold, RFU
LOD run 1	5 CFU/mL	14/20	1.6 (0.8–1.8)	12.9 (5.7–13.8)	4.5–15.1	3.12	137
LOD run 1	1 CFU/mL	10/20	1.2 (0.8–1.8)	9.2 (5.4–13.8)	4.1–14.2	1.85	137
LOD run 1	Human DNA	0/4	–	–	–	1.01	137
LOD run 1	Positive control	3/3	1.8 (0.7–2.1)	13.9 (4.3–15.0)	4.3–15.0	3.25	137
LOD run 1	NTC	0/3	–	–	–	1.0	137
LOD run 2 / patient run B	10 CFU/mL	20/20	1.1 (1.0–1.3)	9.3 (8.6–11.1)	3.7–14.2	6.27	198
LOD run 2 / patient run B	Patient	7/8	0.9 (0.6–1.2)	5.9 (2.9–9.4)	2.7–10.9	6.91	198
LOD run 2 / patient run B	NTC	0/1	–	–	–	1.03	198
LOD run 2 / patient run B	Human DNA	0/1	–	–	–	0.99	198
Patient run A	Patient	4/33	1.0 (0.8–2.0)	9.1 (6.6–13.7)	6.3–14.6	0.99	117
Patient run A	NTC	0/2	–	–	–	0.99	117
Patient run A	Human DNA	0/1	–	–	–	1.0	117
Patient run A	1 CFU/mL	0/1	–	–	–	0.98	117
Patient run A	10 CFU/mL	1/1	4.3 (4.3–4.3)	16.3 (16.3–16.3)	16.3–16.3	1.62	117
Patient run A	100 CFU/mL	1/1	2.7 (2.7–2.7)	15.6 (15.6–15.6)	15.6–15.6	1.94	117
Patient run C	Patient	8/20	0.8 (0.6–1.2)	8.5 (6.5–11.7)	5.0–14.9	1.06	100
Patient run C	NTC	0/1	–	–	–	1.0	100
Patient run C	Human DNA	0/1	–	–	–	1.02	100
Patient run C	Human DNA	0/1	–	–	–	1.04	100
Patient run C	10 CFU/mL	1/1	1.0 (1.0–1.0)	10.7 (10.7–10.7)	10.7–10.7	5.25	100

IQR by the SPSS weighted-average definition; values for positive reactions only. Kruskal–Wallis across 1, 5 and 10 CFU/mL (LOD runs): TTP $p = 0.74$, T50 $p = 0.59$; Mann–Whitney 1 vs. 5 CFU/mL TTP $p = 0.77$.

Table S9. De-identified per-patient results ($n = 52$).

ID	Age	Sex	qPCR result	*E. coli* Cq	Load, CFU/mL	*dxs* copies/mL	LAMP–CRISPR/Cas12a	Positive reactions	TTP, min [a]	Blood culture	SOFA	PCT, ng/mL	Outcome
1	57	M	*E. coli*	39.3	2.7	8.2	Positive	1/1	0.74	Negative	3	1.1	Survival
2	62	F	*E. coli*	36.6	15.3	46.4	Positive	1/1	1.18	Negative	2	52.7	Survival
3	58	M	*E. coli*	38.9	3.5	10.5	Negative	0/2	–	*E. coli*	7	43.0	Survival
4	76	M	*E. coli*	40.3	1.4	4.3	Negative	0/1	–	*E. coli*	0	1.4	Survival
5	57	M	*E. coli*	31.2	466.1	1411.8	Positive	1/1	1.24	*E. coli*	6	233.2	Death
6	75	F	*E. coli*	40.2	1.5	4.6	Positive	1/1	0.81	*E. coli*	1	91.6	Survival
7	64	M	*E. coli*	39.3	2.7	8.3	Positive	1/1	2.34	Negative	2	–	Survival
8	75	M	*E. coli*	39.4	2.6	7.8	Negative	0/1	–	Negative	6	4.6	Death
9	52	F	*E. coli*	39.4	2.6	7.8	Positive	1/1	0.63	*E. coli*	11	224.7	Death
10	69	F	*E. coli*	32.0	280.2	848.7	Positive	1/1	1.31	*E. coli*	7	32.2	Survival
11	98	F	*E. coli*	39.6	2.2	6.8	Negative	0/1	–	Negative	5	0.8	Death
12	82	M	*Streptococcus sp.*	–	–	–	Negative	0/1	–	Negative	1	0.6	Survival
13	82	M	*E. coli*	40.5	1.3	3.8	Negative	0/1	–	Negative	2	0.1	Survival
14	94	M	*E. coli*	37.0	11.6	35.3	Positive	1/2	0.59	*E. coli*	4	–	Death
15	60	M	*Klebsiella pneumoniae*	–	–	–	Negative	0/1	–	Negative	5	1.4	Death
16	41	M	*Klebsiella pneumoniae*	–	–	–	Negative	0/1	–	Negative	2	33.7	Survival
17	20	M	*Klebsiella pneumoniae*	–	–	–	Negative	0/1	–	Negative	0	–	Survival
18	55	M	*Streptococcus sp.*	–	–	–	Negative	0/1	–	Negative	6	0.2	Death
19	65	F	*Acinetobacter baumannii*	–	–	–	Negative	0/1	–	*Klebsiella aerogenes*	4	32.7	Death
20	49	M	*E. coli*	38.5	4.5	13.6	Negative	0/1	–	Negative	4	13.0	Survival
21	53	M	*E. coli*	38.0	6.3	19.0	Positive	1/1	0.85	Negative	14	100.0	Death
22	72	M	*Klebsiella pneumoniae*	–	–	–	Negative	0/1	–	*Klebsiella pneumoniae*	2	91.4	Survival

ID	Age	Sex	qPCR result	*E. coli* Cq	Load, CFU/mL	*dxs* copies/mL	LAMP–CRISPR/Cas12a	Positive reactions	TTP, min [a]	Blood culture	SOFA	PCT, ng/mL	Outcome
23	63	M	*Klebsiella pneumoniae*	–	–	–	Negative	0/1	–	*Staphylococcus aureus*	3	4.4	Death
24	36	M	*Klebsiella pneumoniae*	–	–	–	Negative	0/1	–	Negative	2	–	Survival
25	62	M	*Enterococcus sp.*	–	–	–	Negative	0/1	–	Negative	2	1.1	Survival
26	78	F	*Klebsiella pneumoniae*	–	–	–	Negative	0/1	–	Negative	0	0.1	Survival
27	65	F	*E. coli*	40.0	1.7	5.1	Negative	0/1	–	*E. coli*	8	6.3	Survival
28	68	M	*E. coli*	35.6	28.4	86.0	Positive	1/1	0.62	*E. coli*	5	24.2	Survival
29	78	F	*E. coli*	32.0	280.2	848.7	Positive	1/1	0.92	Negative	5	118.2	Survival
30	81	F	*Enterococcus sp.*	–	–	–	Negative	0/1	–	Negative	0	1.9	Survival
31	71	M	*E. coli; Streptococcus sp.*	37.2	10.3	31.1	Positive	1/1	1.04	*E. coli*	10	388.9	Death
32	81	M	*E. coli*	39.0	3.3	9.9	Negative	0/1	–	*E. coli*	6	43.1	Survival
33	58	M	Negative	–	–	–	Negative	0/1	–	Negative	6	20.0	Survival
34	62	M	Negative	–	–	–	Negative	0/1	–	Negative	0	–	Survival
35	88	M	Negative	–	–	–	Negative	0/1	–	*E. coli*	2	11.6	Survival
36	78	F	Negative	–	–	–	Negative	0/1	–	Negative	5	118.2	Survival
37	51	M	Negative	–	–	–	Negative	0/1	–	Negative	2	1.7	Death
38	100	F	*E. coli*	39.0	3.3	9.9	Negative	0/1	–	Negative	3	100.0	Death
39	59	F	*E. coli*	39.1	3.1	9.3	Negative	0/1	–	Negative	0	–	Survival
40	84	M	*E. coli*	38.9	3.5	10.7	Negative	0/1	–	*E. coli*	0	13.1	Survival
41	67	M	*E. coli*	38.0	6.2	18.7	Positive	1/1	0.99	*E. coli*	4	251.2	Survival
42	83	M	*E. coli*	37.0	11.6	35.3	Positive	1/1	0.65	Negative	0	–	Survival
43	65	F	*E. coli*	36.8	13.2	40.1	Positive	1/1	1.20	*E. coli*	4	294.0	Survival
44	39	F	*E. coli*	40.0	1.7	5.2	Positive	1/1	0.63	Negative	3	20.2	Survival
45	81	M	*E. coli*	40.0	1.7	5.2	Positive	1/2	0.68	*E. coli*	6	14.0	Death
46	62	F	*E. coli*	36.8	13.2	40.1	Positive	1/2	1.17	*E. coli*	5	30.1	Survival
47	77	M	*E. coli*	32.2	246.8	747.3	Positive	1/1	2.52	*Klebsiella pneumoniae*	5	3.0	Death
48	63	M	*E. coli*	39.1	3.1	9.3	Negative	0/1	–	Negative	3	4.2	Survival
49	91	M	*Staphylococcus aureus*	–	–	–	Negative	0/1	–	*Staphylococcus aureus*	5	21.8	Survival
50	89	F	*E. coli*	38.3	5.0	15.2	Negative	0/1	–	Negative	2	12.4	Survival
51	69	M	*E. coli*	40.0	1.7	5.2	Negative	0/1	–	Negative	3	1.1	Death
52	54	F	*Streptococcus pneumoniae*	–	–	–	Negative	0/1	–	*Streptococcus pneumoniae*	7	–	Survival

Patient names, hospital codes and dates were removed. Load from the qPCR master curve; copies/mL with 3.03 *dxs* copies/CFU. [a] Mean TTP of positive reactions. Four patients were tested twice: patient 3, negative in both reactions; patient 14, negative initially and positive on retest; patients 45 and 46, one of two reactions positive. Patients 14, 45 and 46 were classified positive. PCT, procalcitonin; –, not applicable or missing.

Table S10. Agreement between methods for *E. coli* (n = 52): 2 × 2 tables, sensitivity/PPA, specificity/NPA, Cohen's κ and exact McNemar test (IBM SPSS Statistics 20, CROSSTABS).

Index test	Reference	TP	FN	FP	TN	Sensitivity or PPA, % (Wilson 95% CI)	Specificity or NPA, % (Wilson 95% CI)	κ (ASE)	κ 95% CI	McNemar *p*
qPCR	Composite [a]	33	1	0	18	97.1 (85.1–99.5)	100.0 (82.4–100.0)	0.96 (0.041)	0.88–1.00	1.000
LAMP–CRISPR/Cas12a	Composite [a]	19	15	0	18	55.9 (39.5–71.1)	100.0 (82.4–100.0)	0.47 (0.098)	0.27–0.66	< 0.001
Blood culture	Composite [a]	17	17	0	18	50.0 (34.1–65.9)	100.0 (82.4–100.0)	0.41 (0.095)	0.22–0.60	< 0.001
LAMP–CRISPR/Cas12a	qPCR	19	14	0	19	57.6 (40.8–72.8)	100.0 (83.2–100.0)	0.50 (0.099)	0.30–0.69	< 0.001
LAMP–CRISPR/Cas12a, strict rule [b]	qPCR	17	16	0	19	51.5 (35.2–67.5)	100.0 (83.2–100.0)	0.44 (0.097)	0.25–0.63	< 0.001
qPCR	Blood culture	16	1	17	18	94.1 (73.0–99.0)	51.4 (35.6–67.0)	0.37 (0.101)	0.17–0.56	< 0.001
LAMP–CRISPR/Cas12a	Blood culture	11	6	8	27	64.7 (41.3–82.7)	77.1 (61.0–87.9)	0.41 (0.132)	0.15–0.67	0.791

TP, FN, FP, TN relative to the reference. κ 95% CI = κ ± 1.96 × asymptotic standard error (ASE), truncated at 1.00. [a] *E. coli* detected by blood culture or qPCR. [b] All replicate reactions positive.

Table S11. Characteristics of *E. coli* qPCR-positive patients (*n* = 33) by LAMP-CRISPR/Cas12a result and by blood-culture result.

Characteristic	LAMP+ (n = 19)	LAMP− (n = 14)	*p*	Culture+ (n = 16)	Culture− (n = 17)	*p*
E. coli Cq	37.0 (35.6–39.3)	39.1 (38.9–40.0)	0.006	38.4 (36.8–39.8)	39.1 (37.5–39.5)	0.516
E. coli load, CFU/mL	11.6 (2.7–28.4)	3.1 (1.7–3.5)	0.006	4.8 (1.9–13.2)	3.1 (2.4–9.0)	0.516
E. coli load, *dxs* copies/mL	35.3 (8.2–86.0)	9.3 (5.2–10.6)	0.006	14.7 (5.9–40.1)	9.3 (7.3–27.2)	0.516
Age, years	67.0 (57.0–77.0)	75.5 (62.0–85.2)	0.145	68.5 (62.8–79.8)	69.0 (58.0–82.5)	0.971
Male sex	11 (57.9)	9 (64.3)	1.000	10 (62.5)	10 (58.8)	1.000
Diabetes mellitus	6 (31.6)	8 (57.1)	0.173	10 (62.5)	4 (23.5)	0.037
Source: urinary tract	5 (26.3)	4 (28.6)	1.000	5 (31.2)	4 (23.5)	0.708
Source: abdominal	5 (26.3)	5 (35.7)	0.707	6 (37.5)	4 (23.5)	0.465
Source: unknown	7 (36.8)	1 (7.1)	0.098	3 (18.8)	5 (29.4)	0.688
SOFA score	5.0 (3.0–6.0)	3.0 (1.5–6.0)	0.233	5.5 (4.0–7.0)	3.0 (2.0–5.0)	0.042
Septic shock	4 (21.1)	3 (21.4)	1.000	5 (31.2)	2 (11.8)	0.225
WBC, $\times 10^9$/L	14.9 (9.5–19.8)	13.8 (9.8–19.8)	0.827	17.2 (14.1–21.1)	11.3 (5.4–15.2)	0.010
Neutrophils, %	91.3 (83.2–93.3)	86.1 (77.5–91.3)	0.215	91.2 (87.3–93.4)	83.2 (69.5–91.7)	0.020
Platelets, $\times 10^9$/L	127.0 (43.0–199.0)	195.5 (96.5–257.5)	0.063	139.5 (53.5–205.0)	182.0 (106.0–235.5)	0.242
Total bilirubin, μmol/L	22.1 (17.6–53.5)	11.5 (8.5–32.8)	0.025	21.5 (12.6–45.4)	16.7 (10.0–46.8)	0.550
Procalcitonin, ng/mL	72.2 (21.2–231.1)	6.3 (1.3–28.1)	0.004	43.0 (14.0–233.2)	8.5 (1.1–64.5)	0.016
Antibiotics before sampling	7 (38.9)	2 (16.7)	0.249	6 (42.9)	3 (18.8)	0.236
In-hospital death	7 (36.8)	4 (28.6)	0.719	5 (31.2)	6 (35.3)	1.000

Data are n (%) or median (IQR; SPSS weighted-average quartiles); Mann-Whitney *U* test (asymptotic) or Fisher's exact test, unadjusted. Load converted from Cq with the master curve; copies/mL with 3.03 *dxs* copies/CFU.

Supplementary Data Files

Data S1 (Data_S1.xlsx). Sheet *README*; sheet *LAMP_Cas12a_wells*, reaction-level fluorescence, calls, TTP and T50 of the LOD and patient experiments (reaction name and values only); sheet *Specificity_panel*, results for the 55 isolates and controls; sheet *Clinical_deidentified*, the de-identified clinical dataset of the 52 patients used for Tables 7–9, S9, S10 and S11 and qPCR_LOD_Cq, qPCR_dilution_Cq_by_run, qPCR_standard_curves.
