# Supplementary material for: A LAMP–CRISPR/Cas12a Assay for Specific Detection of Escherichia coli in Whole Blood: Analytical Performance and Clinical Evaluation in Suspected Bloodstream Infection
Source: Diagnostics (Basel). 2026 Nov 9;16(21):3692. doi: 10.3390/diagnostics16213692 (PMC13763177; doi:10.3390/diagnostics16213692)
Supplement: Supplementary file 1 [file diagnostics-16-03692-s001.zip › Supplementary_Methods_S1.pdf]

Supplementary Methods S1

Design and in silico validation of the LAMP primer set and the CRISPR/Cas12a guide RNA

S1.1. Target and analysed region

dxs (1-deoxy-D-xylulose-5-phosphate synthase) is a single-copy, chromosomally encoded housekeeping gene, highly conserved within *Escherichia coli* and previously validated as a species-specific target for the direct detection of *E. coli* in blood; all coordinates refer to *E. coli* K-12 substr. MG1655 (GenBank NC_000913.3). In silico analysis used a 650-bp region rather than the full gene, the shortest contiguous stretch containing both the LAMP amplicon and the region targeted by the real-time PCR assay used in this study (Table S1.1).

Table S1.1. Target gene, analysed region and amplicon.

Element	Coordinates (NC_000913.3)	Length	Position in the region	Role
dxs gene	438,315–440,177 (complement)	1,863 bp	—	Target gene, 620 codons
Analysed region	438,545–439,194	650 bp	1–650	Shortest contiguous stretch containing both assays; used for design and in silico validation
LAMP amplicon (F3–B3)	438,921–439,163	243 bp	377–619	Outer amplicon; contains the protospacer and PAM

The design then proceeded in four stages, each constraining the next: the conserved target region was selected; the order of the binding sites was fixed before any sequence was chosen, under the constraint that the Cas12a protospacer and its PAM lie between the F1 and B1 sites, the only segment present in every LAMP product irrespective of concatemer length; candidate sets were generated with PrimerExplorer V5 and evaluated for melting temperature, GC content, free energy at the functional end, inter-site spacing and absence of stable secondary structure; and of the set that satisfied these criteria was selected on the in silico evaluation of Section S1.4 and was the only set synthesised (Table S1.2).

Table S1.2. Oligonucleotides and their design parameters. Strand is relative to the plus strand of NC_000913.3.

Component	Sequence 5′→3′	Strand	Coordinates	Amplicon position [a]	nt	GC %	Tm (°C) [b]	ΔG end [c]
F3	GTCGATGGCGAACAGGACC	+	438,921–438,939	1–19	19	63.2	65.7	−6.42 (3′)
F2	CCACGTCATGCAGCACCTGAT	+	438,956–438,976	36–56	21	57.1	68.2	−4.93 (3′)
LF	CTTTCCTGCAACGCGCCTAT	−	438,978–438,997	58–77	20	55.0	66.2	−4.58 (3′)
F1c	CCATTGTCGCGATTTACT	−	439,001–439,018	81–98	18	44.4	57.7	−5.18 (5′)
crRNA repeat	UAAUUUCUACUAAGUGUAGAU	—	not in the genome	—	21	—	—	—
crRNA spacer	CCGCCGGUCUGGCGAUUGGU	−	439,029–439,048	109–128	20	70.0	—	—
PAM (TTTG)	—	−	439,049–439,052	129–132	4	—	—	—
B1c	GTCACCGCGTGTTGCTCGG	+	439,054–439,072	134–152	19	68.4	69.2	−5.68 (5′)
LB	TAGCGATCCGGGAATTTACGTGA	+	439,090–439,112	170–192	23	47.8	66.7	−6.39 (3′)
B2	GTGAAGGTTCCGGCATGGTC	−	439,119–439,138	199–218	20	60.0	66.2	−6.05 (3′)
B3	CTGATGGCGATTACTCCGG	−	439,145–439,163	225–243	19	57.9	62.7	−7.13 (3′)

[a] Numbered from the 5′ end of F3. [b] Nearest-neighbour calculation at the monovalent-equivalent ionic strength of the LAMP buffer (≈ 358 mM Na^{+})[1], oligonucleotide 0.1 µM. [c] Free energy of the terminal five bases at 37 °C, evaluated at the 3′ end for the priming oligonucleotides and at the 5′ end for F1c and B1c, which do not prime but form the stem. FIP = F1c + F2 (39 nt); BIP = B1c + B2 (39 nt); crRNA = repeat + spacer (41 nt).

S1.3. Guide RNA design

Three constraints fixed the protospacer, applied in order. First, it must lie between the F1 and B1 sites, which leaves a window of 35 nt (439,019–439,053). Second, LbCas12a requires a T-rich protospacer-adjacent motif, originally reported as TTTN [2]; the canonical motif is the TTTV subset, since TTTT supports cleavage poorly. Exactly two 20-nt protospacers with a TTTV motif lie within the window, one on each strand, both preceded by TTTG; the window contains no TTTT motif, so the count is two under either definition and the design space was binary. Third, of the two candidates the more conserved was selected by BLASTn across sequenced *E. coli* genomes. The selected spacer lies within the LAMP amplicon and in the same region of *dxs* as the real-time PCR assay, approximately 250 bp downstream of it.

The protospacer is polymorphic, which accounts for the apparent discrepancy between the 20-nt spacer and the 18-bp perfectly matched span in Table 1 of the main text. Among 4,831 *E. coli* entries with full coverage, the allele matched by the spacer occurs in 2,148 (44.5%), the K-12 allele in 1,857 (38.4%, two mismatches at spacer positions 2 and 5), a third variant in 782 (16.2%) and minor variants in 44 (0.9%). The spacer was designed against the most frequent single allele; because coordinates are reported on the K-12 reference, it matches 18 of 20 positions there. Mismatches at these positions are not expected to abolish *trans*-cleavage: for LbCas12a with a canonical TTTV motif, single substitutions at spacer positions 1–5 permit full cleavage, whereas substitutions at positions 12, 13 and 15 abolish it [3]. That study examined single substitutions only, and we do not extrapolate beyond it.

Two consequences follow.

- A spacer mismatch can only reduce activation of the complex, so it can cause a false-negative but not a false-positive result; the specificity of the read-out does not depend on a perfectly matched spacer.
- The analytical model strain is *E. coli* K-12, which carries both mismatches. The limit of detection, the time to positivity and the whole-blood spiking series were therefore established under the less favourable guide condition.

S1.4. In silico validation

BLASTn (megablast) against the NCBI core_nt database. Unrestricted searches return the 5,000 highest-scoring alignments; because the identity floor of the retrieved set is 96.62% for the 650-bp region and 97.53% for the amplicon, both lists are saturated by *E. coli* and neither reaches *Escherichia fergusonii* or *Salmonella enterica*. Those taxa were therefore examined by taxonomy-restricted search, and no negative conclusion is drawn from absence in an unrestricted list.

Table S1.3. BLASTn validation of the analysed region and of the amplicon. Query coverage was 100% for every alignment listed.

Taxon	Alignments	Identity	Bit score	E value (best)
**650-bp region, unrestricted search (5,000 alignments returned)**				
Escherichia coli (incl. *Escherichia* sp.)	4,789 (95.78%)	96.62–100.00%	1,096–1,173	0.0
Shigella sonnei / *Shigella flexneri*	86 / 40	97.38–100.00%	1,096–1,173	0.0
Shigella dysenteriae / *Shigella boydii*	22 / 21	97.38–98.77%	1,096–1,137	0.0
E. coli laboratory, synthetic, metagenome and vector entries	39	97.38–100.00%	1,096–1,173	0.0
Enterobacter hormaechei [d] / uncultured bacterium	2 / 1	100.00%	1,173	0.0
**650-bp region, search restricted to *Escherichia fergusonii***				
Escherichia fergusonii	76	93.08–95.54%	967–1,042	0.0
**243-bp amplicon, unrestricted search (5,000 alignments returned)**				
Escherichia coli (incl. *Escherichia* sp.)	4,834 (96.68%)	97.53–100.00%	412–439	1×10^{-118}
Shigella sonnei / *Shigella flexneri*	84 / 32	97.53–100.00%	412–439	1×10^{-118}
Shigella dysenteriae / *Shigella boydii*	5 / 3	97.53–98.35%	412–421	3×10^{-113}

E. coli laboratory, synthetic, metagenome and vector entries	39	97.53–100.00%	412–439	1×10^{-118}
Enterobacter hormaechei [d] / uncultured bacterium	2 / 1	100.00%	439	1×10^{-118}
**243-bp amplicon, restricted searches**				
Escherichia fergusonii	76	91.77–96.71%	345–403	3×10^{-111}
Shigella spp.	100	97.94–100.00%	416–439	2×10^{-121}
Salmonella enterica [e]	—	84.71%	270	—
Acinetobacter baumannii / *Homo sapiens* [e]	none	no detectable similarity	—	—

[d] Probable taxonomic misassignment: the species is absent from every other position in both lists, 100% identity over 650 bp is not consistent with the divergence expected between these genera, and the *Enterobacter cloacae* complex isolate in the exclusivity panel was negative by both read-outs. [e] Taxonomy-restricted searches. The output files of every search reported here are archived and available from the corresponding author on request.

In both searches 4,958 of 5,000 alignments (99.16%) belong to the *Escherichia*/*Shigella* clade, and only two entries are assigned to a genus outside it. A second, independent run of the 650-bp search returned the same taxonomic composition and the same identity floor. A total of 1,528 alignments for the region and 1,598 for the amplicon match at 100% identity over 100% coverage. Cross-reaction with *Shigella sonnei* and *Shigella flexneri* is certain, as both match the amplicon and 650-bp region with 100% identity and coverage, with all primer-binding sites, the protospacer and PAM fully conserved; discrimination at this locus is therefore impossible, and the assay is best described as detecting the *E. coli/Shigella* clade rather than *E. coli* sensu stricto. Amplification of *Escherichia fergusonii* cannot be excluded, as locus identity ranges from 91.77–96.71%, and the inner and loop primers remain fully matched, amplification may be delayed rather than prevented, and Cas12a cannot discriminate the resulting product; reliable species-level discrimination would require targeting a different gene.

S1.5. Basis of specificity

An unrestricted search of the 20-nt spacer alone returned 3,148 of 5,000 alignments covering the full 20 nt at 100% identity, of which 98.95% belong to the *Escherichia*/*Shigella* clade; the remaining 33 are unrelated taxa. We do not present this as evidence of specificity. A 20-mer is too short for BLASTn to establish taxonomic specificity: the best possible alignment of a 20-nt query against this database carries an E value of 1.8, and exact 20-nt matches arise by chance in large genomes.

Specificity is architectural, produced by two filters in series. The LAMP reaction restricts amplification, since six binding sites across 243 bp must all be present and correctly spaced; this excludes every organism in Section S1.4 other than *Shigella* spp. and, possibly, *E. fergusonii*. The Cas12a step then restricts the read-out, since only a product carrying the protospacer with its TTTG PAM activates the complex, which is what distinguishes a true amplicon from the primer-driven products that electrophoresis cannot distinguish by morphology. A chance 20-mer match in an unrelated genome is therefore inconsequential, because that genome would first have to be amplified.

S1.6. Experimental verification

Full results are in the main text (Sections 3.1–3.3) and Table S1.

- **Inclusivity.** All 15 *E. coli* isolates were detected. *E. coli* K-12 gave a last/first fluorescence ratio of 8.75 and a time to positivity of 1.79 min, within the range of 3.78–13.85 across the panel, despite carrying both spacer mismatches.
- **Analytical model.** The limit of detection and the whole-blood spiking series used *E. coli* K-12 carried through the complete leukocyte-depletion and extraction workflow, so recovery is included in every reported figure.
- **Exclusivity.** Forty non-target isolates across five organism groups were negative by the Cas12a read-out.
- **Non-specific products:** The non-specific LAMP products observed with human genomic DNA and *Acinetobacter baumannii* were negative by Cas12a read-out, as confirmed by BLASTn, which detected no significant similarity to the human genome or *A. baumannii*; *Salmonella enterica* showed only 84.71% identity, insufficient to support priming by the six-primer set. These findings are consistent with primer-driven non-specific amplification, which has also been reported in no-template controls [4]. Reactions that produced a

LAMP product from non-target or no-template material in this study were negative by the Cas12a read-out, as predicted.

S1.7. Limitations

1. *Shigella* spp. and *E. fergusonii* were not tested experimentally; neither was available as an isolate. For *Shigella* spp. the prediction is as firm as sequence evidence allows; for *E. fergusonii* the outcome is uncertain, since sequence identity does not predict LAMP priming kinetics.
2. Unrestricted BLASTn of this locus is truncated at 5,000 alignments and saturated by *E. coli*; organisms of interest were examined by taxonomy-restricted search instead.
3. core_nt is updated continuously, so the proportions reported are those of the database version searched; they would be expected to shift slightly on repetition, without changing the conclusions.
4. Alignment counts depend on search settings. The restricted *Shigella* search returned 100 alignments and the *E. fergusonii* searches 76 each, at the limits set; these describe the retrieved sets, not the number of genomes in existence.
5. Sequence databases over-represent clinical and laboratory *E. coli*, so the allele frequencies in Section S1.3 describe the sequenced population, not the organisms causing bloodstream infection in a given setting.
6. In silico conservation does not predict amplification efficiency; this is why the exclusivity panel was tested experimentally.
7. The guide targets the 44.5% allele rather than the K-12 allele, and matches 18 of 20 positions against the reference on which coordinates are stated.

References

1. SantaLucia, J. A Unified View of Polymer, Dumbbell, and Oligonucleotide DNA Nearest-Neighbor Thermodynamics. *Proc. Natl. Acad. Sci. USA* **1998**, *95*, 1460–1465. https://doi.org/10.1073/pnas.95.4.1460
2. Zetsche, B.; Gootenberg, J.S.; Abudayyeh, O.O.; Slaymaker, I.M.; Makarova, K.S.; Essletzbichler, P.; Volz, S.E.; Joung, J.; van der Oost, J.; Regev, A.; et al. Cpf1 Is a Single RNA-Guided Endonuclease of a Class 2 CRISPR-Cas System. *Cell* **2015**, *163*, 759–771. https://doi.org/10.1016/j.cell.2015.09.038
3. Misiurina, M.A.; Chirinskaite, A.V.; Fotina, A.S.; Zelinsky, A.A.; Sopova, J.V.; Leonova, E.I. New PAM Improves the Single-Base Specificity of crRNA-Guided LbCas12a Nuclease. *Life* **2022**, *12*, 1927. https://doi.org/10.3390/life12111927
4. Hardinge, P.; Murray, J.A.H. Reduced False Positives and Improved Reporting of Loop-Mediated Isothermal Amplification Using Quenched Fluorescent Primers. *Sci. Rep.* **2019**, *9*, 7400. https://doi.org/10.1038/s41598-019-43817-z
